# “I would really want to know that they had my back”: Transgender women’s perceptions of HIV cure-related research in the United States

**DOI:** 10.1371/journal.pone.0244490

**Published:** 2020-12-31

**Authors:** Tonia Poteat, Anushka Aqil, Dana Corbett, David Evans, Karine Dubé

**Affiliations:** 1 Department of Social Medicine, University of North Carolina School of Medicine, Chapel Hill, North Carolina, United States of America; 2 Department of Health, Behavior, and Society, Johns Hopkins Bloomberg School of Public Health, Baltimore, Maryland, United States of America; 3 Public Health Leadership Program, University of North Carolina Gillings School of Global Public Health, Chapel Hill, North Carolina, United States of America; 4 Delaney AIDS Research Enterprise (DARE) Community Advisory Board, New York City, New York, United States of America; Simon Fraser University, CANADA

## Abstract

Forty-four percent of Black transgender women are living with HIV, and many face challenges with HIV care engagement. An HIV cure has much to offer this population, however little HIV cure-related research has included them. We conducted 19 face-to-face in-depth interviews with 10 Black transgender women living with HIV. Interviews were audio recorded, transcribed verbatim, coded, and analyzed using content analysis. Our interview guide contained three categories: 1) perceptions of HIV cure-related research and participation, 2) perceptions of HIV treatment and treatment interruptions, and 3) considerations for transgender women and HIV cure-related research. Salient themes included skepticism about HIV cure strategies and limited benefits compared with an undetectable viral load. Willingness to interrupt HIV treatment for research was low and linked to being able to go back on the same HIV treatment without consequence when the study ended. Concerns about being a test subject and perceptions of risks versus benefits of various strategies also affected willingness to take part in HIV cure-related research. Centering the dignity and autonomy of research participants as well as building upon and supporting existing social networks were identified as important facilitators for engaging Black transgender women in HIV cure-related research. Specific to Black transgender women, other concerns included the desire for gender-affirming research staff, community-building among transgender women, and safety issues associated with risk of transphobic violence when traveling to study visits. Participants stressed the importance of HIV cure-related researchers providing accessible and complete information and expressing genuine care and concern for transgender communities.

## Introduction

With an estimated HIV prevalence of 44%, Black transgender women are the most heavily impacted population in the US [1]. A growing body of research indicates that Black transgender women living with HIV face significant barriers to engagement along the HIV care continuum [2, 3]. Even when engaged in HIV care, Black transgender women frequently experience antiretroviral treatment (ART) interruptions [4] and adherence challenges [5] that present barriers to health benefits of durable viral suppression. Incarceration, homelessness, substance use, violence victimization, and prioritization of gender affirming care have been identified as barriers to viral suppression among Black transgender women [3].Given the high proportion of Black transgender women living with HIV and the challenges they face in achieving the benefits of ART, an HIV cure has much to offer this population. However, a recent systematic review found that only one transgender woman participated in an HIV cure-related study to date [6].

Despite remarkable advances in the treatment of HIV, two individuals–Timothy Ray Brown (or the Berlin patient) and Adam Castillejo (the London patient), have ever achieved a durable cure [7, 8]. In recent years, the U.S. National Institutes of Health (NIH), private industry and foundations have increased investment in HIV cure-related research [9, 10]. Current approaches attempt to achieve either a complete cure which would eliminate all replication-competent forms of HIV or a sustained ART-free suppression in which the virus would not be completely eliminated [11]. Examples of HIV cure-related research approaches include latency-reversing agents (e.g., awakening dormant HIV to make it visible to the immune system), gene therapy and stem cell transplants (e.g. making cells resistant to HIV infection), and immune-based strategies or therapeutic vaccines (e.g. strengthening a person’s immune system). These HIV cure-related research interventions typically involve high clinical risks and limited personal benefits to individual study participants [12, 13]. Some HIV cure-related research protocols also require the interruption of highly effective HIV treatment to demonstrate whether an intervention had the intended effect. HIV treatment interruptions further carry clinical and emotional risks to HIV cure trial participants as well HIV acquisition risk to their sexual partners [14].

In order to assess efficacy, HIV cure-related research requires people living with HIV to participate in studies. Data on the perspectives of people with HIV on current HIV cure-related research and their willingness to participate is limited. Existing studies suggest that many people with HIV may be reluctant to engage in HIV cure research due to concerns about risks such as pain, antiretroviral resistance, and worsening HIV [15, 16]. Altruism, stigma reduction, and the potential to be free from concerns about HIV transmission to others were common motivators for participation [15–18]. Quantitative data suggest that Black people and women of any race were less willing to participte in biomedical HIV cure-related research [19].

Even though Black transgender women are heavily HIV-impacted, we found only one study, recently published, that assessed barriers to and facilitators of transgender women’s HIV research participation [20]. Barriers identified during online focus groups included limited research opportunities, medical mistrust, fear of mistreatment, concerns about safety and confidentiality, HIV stigma, and competing priorities. Facilitators were peer involvement, incentives, flexibility and choices, multiple modalities and methods, and centering of transgender people. While those findings are informative, that study focused on survey research, not biomedical intervention research. We found no published studies assessing Black transgender perspectives specific to current HIV cure-related research strategies.

This exploratory study sought to address the gap in knowledge by eliciting Black transgender women’s opinions of HIV cure-related research and factors that may affect their willingness to take part in these studies. Specific objectives included exploring participants’ perceptions, attitudes, and understandings of HIV cure-related studies, with an emphasis on identifying perceived risks, benefits, facilitators, and barriers to participation. We also sought participants’ recommendations to facilitate implementation of these studies in a way that is inclusive of Black transgender women.

## Materials and methods

### Reflexivity

The study team was diverse along lines of race, gender, sexual orientation, and HIV status. No member of the team identified as a Black transgender woman. However, two investigators have collaborated with many Black transgender women as part of community engaged research and advocacy efforts over the course of many years. Two other investigators have many years of conducting community engaged research on HIV cure. All investigators have a strong commitment to community engagement and amplifying the voices research participants.

### Study design, setting, eligibility, recruitment, and data collection

This exploratory qualitative study used sequential in-depth interviews with Black transgender women. We used flyers to recruit participants from local HIV clinics. A member of the study team (A.A.) screened interested participants for eligibility by telephone before scheduling their first interview. Between July and August 2018, A.A conducted 19 face-to-face in-depth interviews with 10 Black transgender women living with HIV in a private research office in Baltimore, Maryland (with one exception, each participant took part in two interviews.). Eligibility criteria included being aged 18 years or older, male-assigned at birth, identifying as a woman or transgender woman, and self-reported positive HIV status. The study was approved by the Institutional Review Boards at the University of North Carolina at Chapel Hill and the John Hopkins Bloomberg School of Public Health. Participants were recruited via word-of-mouth and flyers posted at health care centers in Baltimore. In general, the first interview addressed perspectives on and barriers and facilitators to HIV cure-related research. The second interview focused on perceptions of risk and benefits for three broad HIV cure-related research strategies. The full interview guides are available as supplementary files (S1 Appendix). Participants were provided with a brief description of three broad approaches to HIV cure-related research approaches adapted from The Well Project website [21], including: 1) latency reversing agents; 2) gene therapy and stem cell transplants; and 3) therapeutic vaccines. Each interview lasted between 30–90 minutes, with an average duration of one hour. Discussions were focused by the use of semi-structured interview guides. Participants received $50 USD for each interview.

### Coding and analysis

Interviews were audio recorded and transcribed verbatim by a professional transcription company, then coded in MAXQDA by one coder (K.D.) using *a priori* codes derived from the interview guides. Coded textual data were extracted into Excel spreadsheets and read iteratively across and within participants by three investigators (T.P., K.D., and D.C.) using conventional content analysis to identify categories of codes, patterns across codes, and recurrent themes [22, 23]. Differences in interpretation of coded themes were discussed and resolved by consensus.

### Ethics statement

Our qualitative study was approved by the Institutional Review Boards (IRBs) of John Hopkins and the University of North Carolina at Chapel Hill. Both IRBs approved provision of verbal informed consent from our study participants in order to reduce the risk for loss of confidentiality. Verbal informed consent was documented on each participant’s interview transcript. We followed strict confidentiality guidelines to mitigate safety concerns for our participants.

## Results

### Participant characteristics

The age of participants ranged from 30–71 years old with a mean of 45 years. Most participants had been living with HIV for 10 years or more and all were engaged in HIV care, taking ART, and had a self-reported undetectable viral load. Nine participants had a high school education or higher, although none had completed college. Six received social security or disability income (**Table 1**).

**Table 1:** Demographic characteristics of transgender women participants in HIV cure-related in-depth interviews (Baltimore, MD– 2018).

Age (years)	N (%)
18–39	3(30)
40–49	4 (40)
50–79	3 (30)
**Educational Attainment**	
Some College or trade school	4 (40)
High school graduate or less	6 (60)
**Source of Income**	
Work	2 (20)
SSI/Disability/Food Stamps	8 (80)
**Time Since HIV Diagnosis (years)**	
≤10 years	2 (20)
>10 years	8 (80)
**Seen for HIV Care in past 6 months?**	
Yes	10 (100)
No	0 (0)
**Currently on ART?**	
Yes	10 (100)
No	0 (0)
**Viral Load Undetectable?**	
Yes	10 (100)
No	0 (0)
**Completed Parts 1 & 2**	
Yes	9 (90)
No	1 (10)

### Summary of results

Results were grouped into three overarching categories based on our interview guide: 1) perceptions of HIV cure-related research and participation; 2) perceptions of HIV treatment and treatment interruptions; and 3) considerations for Black transgender women and HIV cure-related research. Emergent themes within each group are listed in **Table 2** and outlined below with illustrative quotes. Additional quotes are available as supplementary material (S1 Table), also organized by theme.

**Table 2:** Summary of themes with illustrative quotes from in-depth interviews about HIV cure-related research with transgender women participants (Baltimore, MD– 2018).

**Perceptions of HIV Cure-Related Research**	
**Meanings of HIV Cure**	*“You're rid of it just like if you had some type of—like syphilis*. *Not syphilis but something like chlamydia or something*, *and you just take the medicine and it clears up and it's gone*.*”*–Participant 003, Interview 2
	*“Make sure that it works on everybody that’s on every medicine*, *not just people who are on one pill or three pills or for transgender or for gay males*. *It has to work on everybody*.*”*–Participant 009, Interview 1
	*“I mean*, *not having to take pills every day*. *I think of being able to love without something in between us*. *You know what I mean*? *I will never know what he feels like*.*”*–Participant 002, Interview 1
**Undetectable HIV Status versus ‘Cure’**	*“Undetectable*, *to me*, *is that I still have it*, *I cannot pass it on if I’m undetectable*, *but I have to take my medication*… *Cured’ would be I don’t have to take my medication anymore*. *I wouldn’t have to take my medication anymore*, *but I would have to have safe sex*.*”*–Participant 001, Interview 1
**Cure optimism**	*“I think they're getting close*. *Because the medicines*, *they didn't have these good medicines at first*. *As soon as you found out you had it*, *the next week they would die*. *(…) I think they're getting close because if they're doing this good with the medicines then they're on the right track*.*”–*Participant 003, Interview 1
	*“I really*, *truly don't think there's a cure*, *but I think they're really*, *really*, *really close to finding one*. *And if they have*, *it's been a hush-hush issue*.*”*–Participant 001, Interview 1
**Cure skepticism**	*“Because a lot of people think that it's never going to hit them and there's no possibility*. *And if it is a possibility*, *if it is already here*, *that they'll never be able to afford it*.*”*–Participant 006, Interview 1
	“*I don't know why it's so hard for them to find—they can find a cure for everything but they can't find a cure for that*. *I don't know why it's so hard*.”–Participant 007, Interview 2
	*“If you don't take your medicine*, *it can come back stronger and harder*.*”*–Participant 002, Interview 2
	*“They keep saying remission*. *What do it mean*? *It keep coming back*?*”*–Participant 007, Interview 2
**Perceptions of HIV Research**	*“I would be dead right now if people hadn't did research*. *That's why research is–it's very important*.*”*–Participant 001, Interview 1
	*“I think when you hear the word research you think experiment*. *You think that the person don't know what they doing (…) I think people think of it as the researchers are dissecting you to try to make a plan or something*. *So I think most people are just fearful of the word research*.*”*–Participant 005, Interview 1
**Reasons not to participate**	*“For me to just up and change and do something different*, *I would have to have a lot of faith in what I was being presented*. *(…) I don't think I could take another body change*. *And my grandfather always told me*, *"If it ain't broke*, *don't fix it*.*" So*, *yeah*. *I just couldn't*. *It would feel like betrayal in a way*.*”*–Participant 002, Interview 2
	*“Suppose it's not going to work*, *and now they've boosted your count and weakened your system*. *And their plan didn't work*, *so now you're just at a worse standpoint than you started*. *(…) I definitely wouldn’t do* [research].”–Participant 006, Interview 2
	*“I don't know where to go to get into them*. *I don't have the information or whatever to get into those kind of researches*.*”*–Participant 003, Interview 1
	“*Oh*, *now*, *transportation could be a problem*.*”*–Participant 001, Interview 1
**Reasons to participate in HIV cure-related research**	*“I'll go and participate because I figure the more people that they can get information from*, *the better*, *especially in dealing with the transgender community*.*”*–Participant 002, Interview 1
	*“If I can do something to help prolong somebody else's life*, *even though I might leave mine in the process*, *I would do it*.*”–*Participant 001, Interview 1
	*“I'm going because I want to see a better outcome with this*. *You know what I mean*? *I want to see a better outcome*. *I don't want men like my boyfriend to fall prey to HIV or possibly catch AIDS*.*”*–Participant 002, Interview 2
	*“So I think that a lot of transgender girls look for opportunities that would help us*. *You know what I mean*? *To be a little bit more independent*. *So that's why these studies that come along*, *a lot of girls do attend because it's how they stay connected in their own way*.*”*–Participant 002, Interview 1
**Perceived benefits of HIV cure-related research**	*“The benefits would be better health*.*”*–Participant 009, Interview 2
	*“The person actually participating is not benefiting*. *They're helping somebody else benefit off of what they get*.*”*–Participant 008, Interview 2
	*“The fame*, *all that*. *Because you're probably famous*. *You're really going to be famous*, *and you really want to be rich*. *So there you go*.*”*–Participant 003, Interview 2
**Perceived risks of HIV cure-related studies**	*“Death*. *Sickness*. *(…) Other infections or reaction to it*. *Whatever it is*. *However they try and do it*. *(…) Being taken off your medicine and your virus mutating*. *Not being able to take that medicine you were taking*.*”*–Participant 009, Interview 2
	*“The risk*, *I think*, *is that some people's bodies might reject their medication and they might end up dying from something that they're trying to help you to care for*. *I mean*, *I guess that's part of research*.*”–*Participant 001, Interview 2
	*“I don't know if it can mess up what's going on inside your body or you got to worry about the side effects and all that other stuff*. *I mean*, *I'm all for it but I don't want to be the guinea pig*.*”*–Participant 007, Interview 2
	Side effects
	*“Yeah*, *the side effects*, *mainly the side effects because—and side effects cover a lot of things*, *mentally*, *physically*, *emotionally*.*”*–Participant 002, Interview 1
	*“Like life-threatening side effects like fevers and heart problems and seizures and stuff like that*. *That would be scary*.*”–*Participant 009, Interview 1
	*“I don't know*. *That's kind of hard to say*. *Because all of it sounds dangerous*. *And when it comes to the approach of something new*, *now that they came up with*, *that's trying to do something*, *because you don't know the side effects of it*.*”*–Participant 009, Interview 2
**Perceptions of unacceptable risk in HIV cure-related studies**	*“Let's hope I don't grow another eye on my forehead*.*”*–Participant 002, Interview 2
	*“Anything besides death I'll deal with*. *I'll deal with a little headache and constipation and all that*, *but if it says death then I'm not dealing with this*.*”*–Participant 003, Interview 2
	*“Depression comes along with this*, *and depression makes you feel like everything is against you*. *Everything is against you*. *Like you're not going to win*. *You're at the finish line*, *you've had the training*, *you still didn't win*. *And that's depression*.*”–*Participant 006, Interview 2
	*“There's none*, *"too much of a risk"*. *Because*, *whatever's going on*, *they're going to make sure*, *if there's a vaccine or they're using needles*, *they're going to make sure I'm protected*.*”–*Participant 004, Interview 2
**Concerns about HIV cure-related studies**	*“I ain't cool being a test subject*. *Meaning like*, *you shooting a vaccine into me*, *but do you know what it is that you're actually doing*? *Or what it is*? *Because once you put it in you can't just take it out as easy as you put it in*.*”–*Participant 008, Interview 2
	*“There are no concerns*. *None*, *because I'm helping someone—I'm helping myself*. *I'm helping […]*. *I'm helping […]*. *I'm helping […]*. *Whatever*, *whatever*. *It's to help people*. *We're put on this earth to help people*. *We're not supposed to be selfish*.*”*–Participant 004, Interview 2
**Perceived burdens of HIV cure-related studies**	*“Not knowing what's being done to you*.*”*–Participant 008, Interview 2
	*“Time-consuming and—I'd say*, *basically*, *time-consuming*.*”*–Participant 009, Interview 2
	*“The traveling*. *The traveling is really bad*. *Well*, *for certain people that's outside the area*.*”*–Participant 002, Interview 2
**Personal impacts of an HIV cure**	*“It would get better*. *I mean*, *I wouldn't have to worry about taking a pill every day now or any of that*.*”*–Participant 009, Interview 1
	*“I would be careful of the people I picked as sexual partners*. *I would be careful on how I treat people*. *I would* [have] *so much gratefulness that I would have to get into something that was helping somebody else*. *That would push me just to make people's lives or things better*. *If there was a cure and I got the cure*, *and I was able to be cured from HIV*, *everything would change*.*”*–Participant 005, Interview 1
	*“If I could get cured and got rid of this virus I would be scared to have sex*. *Because I wouldn't want to–I know what I've been through*. *I wouldn't want to have sex*. *I would want to have sex but I wouldn't have sex*.*”*–Participant 001, Interview 2
**Community impacts of an HIV cure**	*“I think that it would be a good thing for the trans community*. *I think that a lot of people would wake up from the bitterness and the hurt*, *and the discrimination*, *and everything negative that they had to go through*, *it wouldn't matter no more*. *(…) So if somebody offered you the gift of the cure them things that bothered you before*, *it can't really bother you like that again because it's like you got a second chance at life*.*”*–Participant 005, Interview 1
	*“Everybody would try to get it*, *and then they would think because oh*, *there's a cure*, *they're going to continue to have unprotected sex and don't worry about catching it again because they got a cure*.*”*–Participant 009, Interview 1
	*“I think research got to be done to make a cure but once you accomplished the cure*, *then what*? *(…) Is you going to keep it*? *Is you going to try to figure out how can you (…) get money off it*? *Or is you just going to give it to people because they need it*?*”*–Participant 008, Interview 1
**Overall impression of HIV cure-related research strategies**	*“It's magnificent and fascinating how advanced science is*, *wow*.*”–*Participant 004, Interview 2
	*“Well*, *how long have they been doing these researches like this*? *(*.* *.* *.*) If they've been trying it this way for all these years*, *I mean maybe they need to try something else*. *Because if they've just been working at it since the '80s on this certain situation and no results yet*, *then they need to try to aim everything a different way*.*”*–Participant 003, Interview 2
	*“When you go to dialysis*, *don't they flush your blood*? *They take impurities and stuff out your blood*. *So*, *why can't they do it that way*?*”*–Participant 006, Interview 2
	*“I would do all of them*. *(…) Because if one don't work*, *go to the next one*. *(…) I'm not afraid of basically trying anything when it comes to that*. *And it would help other people out*. *Because people helped me out*. *They get me where I'm at now*.*”*–Participant 001, Interview 2
	*“So*, *truthfully*, *none of those would work for me because I'm focused on where I am right now*. *Unless I see a phenomenal turnaround*, *I'd just rather stay safe where I'm at*.*”*–Participant 002, Interview 2
**Perceptions of latency reversing agents**	*“What if it's a lot of them that's hiding [in HIV reservoirs]*? *What if they all decide to come out and then kill you*? *(…) That sounds like that person’s going to be real ill*. *They’re going to be real sick*.*”–*Participant 006, Interview 2
	*“I wonder if the viruses—the bugs that you can't find but you wake them up*. *Will they start reproducing*?*”–*Participant 001, Interview 2
	*“The part that keeps triggering my mind*, *when it wakes up*, *where it's going to be going*?*”–*Participant 007, Interview 2
	*“It feels like I’m gambling at the casino with that one*. *(…) I come for an appointment*, *they say*, *‘Oh*, *well*. *We have to change it this time because it's constantly changing now*,*’ or*, *‘We found it*,*’ or*, *‘One was hiding somewhere*, *now it didn't spread–‘ no*. *That's putting me on edge because I'm wondering*, *‘Is this going to work*? *Is this going to pull me through‴*–Participant 002, Interview 2
	*“You don't really know what it's killing*, *when it's killing*. *Rather just say you're not only think it's killing something*, *then you wind up having brain damage or something like that*. *If the pill or whatever it is activates itself*, *then how can you stop it*?*”–*Participant 008, Interview 2
**Perceptions of gene therapy and stem cell transplants**	*“Where is your DNA on the inside*? *Where*?*”*–Participant 007, Interview 2
	*“Okay*. *That’s a little scary*.*”*–Participant 006, Interview 2
	*“Wouldn't that be painful*? *Because that's like taking blood out and putting more blood in*.*”–*Participant 010, Interview 2
	Interviewer: *“Okay*. *And then so you're saying the gene therapy seems the riskiest*?*”*
	Participant: *“Yeah*. *Because it's messing with the genetic code*.*”–*Participant 009, Interview 2
	*“So it's completely like going into a dark room*. *You don't know if it's in there right*.*”–*Participant 001, Interview 2
	*“I think that I would probably go through with the putting a gene in my body*. *The stem cell*. *And then trying to boost my immune system up*. *I don't get sick much*, *but that would be a good thing*. *I wouldn't have to worry about catching any colds or anything*.*”–*Participant 001, Interview 2
	*“Okay*. *The safest option might be number two* [gene therapy]. *(…) Because you can learn about the genes*, *you can know more about the people's body or whoever the patient is*. *That way you can work your way into knowing how to treat things*. *That seems to be easier and less complicated*.*”–*Participant 010, Interview 2
**Perceptions of therapeutic vaccination strategies**	*“That’s just like a shield*, *right*? *(…) Okay*, *it sounds crazy*, *but it's like Truvada with a sword (…) It sounds like a raging war inside my body*.*”*–Participant 006, Interview 2
	*“I would be scared of the vaccinations*. *(…) Because I mean*, *you’d get so sick*. *(…) I think that is very dangerous*.*”–*Participant 001, Interview 2
	*“You take for instance*, *when the H1N1 flu and all that came out*, *I never got the flu shot*. *Never took the H1N1*. *So one year I'm just like*, *‘Okay*. *I'm going to take it*.*’ The worst thing I ever did*. *I was sick for three weeks*. *I said*, *‘I'm never taking that shot again*.*’ So that would be my support and evidence*. *I went through this before*.*”*–Participant 008, Interview 2
	*“Does the infection fight back*? *Don’t it fight back*? *That’s when it mutates and stuff*. *(…) So suppose they shoot you with the shot*, *and the strain that you carry is stronger than—and what it if it takes you down*? *(…) That’s just my concern with the vaccine*.*”*–Participant 006, Interview 2
	*“By there being so many different strands*, *would there be different vaccines you can get or would there just be one that they're trying to make that work*? *For all the different strands of HIV*? *(…) I would say that would be kind of hard*, *really*.*”*–Participant 001, Interview 2
	*“The [vaccine approach] is just a lie because a vaccine whether it's a pill*, *whether it's a egg shot or whatever*, *they shoot you with something*. *So*.*”*- Participant 008, Interview 2
**Perceptions of HIV Treatment and Treatment Interruptions**	
**Perceived advantages of HIV medications**	*“The medicine has really gave [sic] me an opportunity to live*. *You know what I mean*? *I've gained weight*. *It helped me to eat*. *I even get a little rest*. *You know what I mean*? *It's really changed my life*. *I don't have the depression that I used to have*.*”–*Participant 002, Interview 2
	*“Well*, *nothing's bad about it because I don't get side effects from it*. *And what's good about it is that I feel normally healthy*, *and I'm not sick*, *and I'm undetectable*. *and it just keeps me healthy*.*”*–Participant 009, Interview 2
	*“The good thing to me about it is that I have to just take one little tiny pill*.*”*–Participant 007, Interview 2
**Perceived drawbacks of HIV medications**	*“Once you do something long enough*, *it becomes natural*. *But it really shouldn't be a natural thing of life to get up every day and take a pill to survive*. *That's not how life's supposed to be*.*”*–Participant 002, Interview 2
	*“It's a constant reminder*. *A constant reminder*. *I try to just chuck a handful of my pills*. *I take hormone pills*, *and I take testosterone blockers*. *So I just chuck them all*, *yeah*.*”*–Participant 006, Part 2
**Willingness to interrupt HIV treatment**	*“I don't think it would be bad because I would be monitored*. *If things start going bad*, *then they would probably know what to do*, *I hope*.*”*–Participant 010, Interview 1
	*“It depends on how long I would have to stop*. *I don't know*, *gosh*. *The longest time I would stop would be for a month*.*”*–Participant 001, Interview 1
	*“I would do it for a study*. *Yeah because like I said I can always get back on*.*”–*Participant 003, Interview 1
**Concerns about HIV treatment interruptions**	*“And if there’s any change in—with the medicine that I’m taking*, *will I be able to go back to my medicine again*.*”*–Participant 002, Interview 1
	*“They don’t really have that many medicines out here*. *It’s hard to find medicine that your body’s going to work with if you keep messing up all the medicine that they give you*, *and some have ran out of options*.*”*–Participant 009, Interview 1
	*“I would be very fearful of stopping my HIV medicine because I know how fast the virus can grow*. *(…) How long would I have to stop it*? *And if I do stop it whatever research you're all doing to find this cure*, *what are these drugs going to do to me*? *Are they going to make me sick*?*”*–Participant 005, Interview 1
**Considerations for Transgender women and HIV cure-related Research**	
**Competing Priorities for transgender women**	*“Now that that cure word is mentioned*, *that would be number one*. *That would be the first thing on the list*.*”*–Participant 005, Interview 1
	*“It’s not a priority*, *no*. *(…) Because people can live with HIV and live their full lifespan and die from natural causes and not from the disease*.*”*–Participant 009, Interview 1
	*“Right now*, *I really don't care because I'm still going to be cautious*. *I'm still going to have safe sex when I do decide to have it*. *And it's not going to make me run out and say*, *hey*, *boys I'm free*. *No*. *That's not going to work*.*”*–Participant 010, Interview 1 & 2
**Necessary protections for HIV cure-related studies**	*“Be honest*. *It won't take much*. *You know what I mean*? *If you're doing something and there's a choice*, *I think that it's very important to always make sure that a person know that they have a choice*. *(…) The ethical thing to do is to always make sure that person has a voice and that you're being honest*. *(…) Because if something bad just starts happening and you didn't tell me about it*, *but you knew it was going to happen*, *there is no ethics in that*.*”*–Participant 002, Interview 2
	*“Have someone that's gone through it before you that could give you their experience (…) I think that would be another way to sooth the mind of someone that doesn't know what to expect*.*”*–Participant 005, Interview 1
	*“I would really want to know that they had my back*. *If something went wrong*, *are you going to make sure that people know that I'm doing this*, *or I was in this situation*? *(…) Are you willing to make sure*
	*I'm okay because I decided to try something that was supposed to benefit everybody else*?*”–*Participant 002, Interview 1
	*“Paid compensation*. *Extra healthcare*. *24/7 awareness*. *When I say that*, *I mean*, *constant checkup*. *Okay*? *Constant checkup*. *And back to the privacy*. *Confidential*. *As long as it's confidential*.*”*–Participant 006, Interview 1
	*“You have someone's life in your hand*. *You're controlling someone else's health*. *(…) You have to worry about that person who you are experimenting on*. *And that's what I think most researchers should really consider*. *I'm just not a cadaver or–I'm a living person*.*”*–Participant 001, Interview 2
	“*Have evidence that what they're trying to do has a possibility of it working (…) Evidence*. *Okay*? *Not*, *‘I'll take it and try this one*, *and then try this one*, *and try this one*.*’ Nope*. *(…) I don't want to go through that*.*”*–Participant 006, Interview 2
**Considerations specific for transgender women**	*“I've been to an interview before*, *and they were using ‘He’ (…) that it was really uncomfortable because it was a man*. *And that was like—I was like*, *‘I just can't do this one*.*’ He referred to us as*, *‘He*.*’”*–Participant 002, Interview 1
	*“It's just we're all participating as humans*, *not as different genders or races or anything*, *just as a person that's trying to help find a cure*… *It shouldn't be like trans-women should be singled out*. *We should be just a candidate just like anyone else*.*”*–Participant 003, Interview 1
	*“Their personalities*. *Their lifestyle*. *(…) All of them got different personalities*. *And*, *it's just like meeting different people*. *We all take information differently*. *And some of them feel like*, *"Oh*. *It's too personal*. *This and that*.*" And lifestyle is like acceptance of who they are and not having them feel judged*, *us feel judged*. *That's it*.*”*–Participant 009, Interview 2
**Information for transgender women**	*“All that's available*. *Everything that they know*, *we should know*. *Not how it's done*, *but why it's done and when it's done and the cause and side effects and all*, *everything*. *All the information*.*”*–Participant 009, Interview 2
	*“I think they should get information of how it's coming along with the study*. *I participated in so many studies*, *but I never know where they go from there*. *You know what I mean*? *(…) once you've got up enough data that you can figure—at least let us know what's going on*.*”*–Participant 002, Interview 2
**Strategies to encourage participation of transgender women in HIV cure-related research**	*“Go into these streets and these areas where they be lingering and being at and social events that they be having out in the cities like Pride*. *And our state*, *but all these other little festivals*. *And handing out flyers or posting things on social media*, *where a lot of people be at*.*”*–Participant 009, Interview 2
	*“Just because you call us in to say*, *"Oh*, *come get this $50 and do this thing*,*" that doesn't help us communicate with each other or know what's going on within our community*, *(…) So if y'all are so interested in making sure that we're good*, *pull us together and let's have a community powwow of a transgender meeting*. *And y'all can let us know where you're coming from*, *and we can let you know where we're coming from*.*”*–Participant 002, Interview 2
	*“I don't think that it should be just for trans*. *I think that it should be for the LGBT–the whole community (…) but it needs to be in a group setting (…) with different settings of people*.*”–*Participant 006, Interview 2
	*“Except*, *especially in the evening*, *most studies need to have little cab vouchers or some tokens for the people*. *Because when you give them the card*, *sometimes when you give them the cash*, *you can't just go out and get change*. *Not anymore*. *It's not safe*. *So it's taking all of that into consideration*.*”*–Participant 010, Interviews 1 & 2

### Part 1: Perceptions of HIV cure-related research and participation

#### Meanings of HIV cure

Participants understood an HIV cure to mean that people living with HIV (PLWHIV) could stop taking a daily medication and their bodies would be completely free of the virus, i.e. an ‘eradicating’ cure. One participant emphasized the requirement that a cure would work for everyone regardless of sexual orientation, gender, and race. Some participants expressed that being cured of HIV would provide them with enhanced sexual intimacy since they would no longer have to use condoms.

*“You're rid of it just like if you had some type of–like syphilis*. *Not syphilis but something like chlamydia or something*, *and you just take the medicine and it clears up and it's gone*.*”*–Participant 003, Interview 2

*“Make sure that it works on everybody that’s on every medicine*, *not just people who are on one pill or three pills or for transgender or for gay males*. *It has to work on everybody*.*”*–Participant 009, Interview 1

*“I mean*, *not having to take pills every day*. *I think of being able to love without something in between us*. *You know what I mean*? *I will never know what he feels like*.*”–*Participant 002, Interview 1

#### Undetectable HIV status versus ‘cure’

Participants understood the main difference between HIV cure and undetectable status to be that for the former they would no longer have to take daily medication. Those who knew that HIV cannot be transmitted by someone with an undetectable viral load understood that for HIV cure and undetectable status alike, HIV should not be transmitted between partners. However, several participants mentioned the need to continue condom use after an HIV cure since they could still acquire other sexually transmitted infections and would again become vulnerable to HIV infection.

*“Undetectable*, *to me*, *is that I still have it*, *I cannot pass it on if I’m undetectable*, *but I have to take my medication*… *‘Cured’ would be I don’t have to take my medication anymore*. *I wouldn’t have to take my medication anymore*, *but I would have to have safe sex*.*”*–Participant 001, Interview 1

#### Cure optimism

Some participants expressed much optimism about the discovery of an HIV cure. Improvements in HIV treatment medications, in particular, have instilled hope that a cure is soon to come. While most participants did not believe that a cure has already been discovered, some asserted that a cure does exist and is being hidden from the general public, reserved for only certain members of the population.

*“I think they're getting close*. *Because the medicines*, *they didn't have these good medicines at first*. *As soon as you found out you had it [HIV]*, *the next week they would die*. *(…) I think they're getting close because if they're doing this good with the medicines then they're on the right track*.*”–*Participant 003, Interview 1

*“I really*, *truly don't think there's a cure*, *but I think they're really*, *really*, *really close to finding one*. *And if they have*, *it's been a hush-hush issue*.*”*–Participant 001, Interview 1

#### Cure skepticism

Other participants were pessimistic that a cure will be discovered; and they felt that if one is discovered, it will be too expensive to access. Limited understanding of the challenges to finding an HIV cure fueled much of this pessimism and frustration.

*“Because a lot of people think that it's never going to hit them and there's no possibility*. *And if it is a possibility*, *if it is already here*, *that they'll never be able to afford it*.*”–*Participant 006, Interview 1

*“I don't know why it's so hard for them to find–they can find a cure for everything but they can't find a cure for that*. *I don't know why it's so hard*.*”–*Participant 007, Interview 2

Some participants expressed skepticism about specific cure approaches which would not completely rid the body of the virus, such as a ‘functional cure’ or remission.

*“If you don't take your medicine*, *it can come back stronger and harder*.*”*–Participant 002, Interview 2

*“They keep saying remission*. *What do it mean*? *It keep coming back*?*”*–Participant 007, Interview 2

#### Perceptions of HIV research

Perceptions of HIV research ranged from an appreciation of benefits of scientific research to fear of being treated as a test subject. Research was sometimes viewed as a positive way to learn and improve medicines and at other times depicted as a disorganized trial-and-error process at the participants’ expense.

*“I would be dead right now if people hadn't did research*. *That's why research is–it's very important*.*”*–Participant 001, Interview 1

*“I think when you hear the word research you think experiment*. *You think that the person don't know what they doing (…) I think people think of it as the researchers are dissecting you to try to make a plan or something*. *So I think most people are just fearful of the word research*.*”–*Participant 005, Interview 1

#### Reasons not to participate

Participants expressed a variety of reasons why they would not participate in HIV cure research. Some participants described having an effective antiretroviral regimen and not wanting to jeopardize their good health for the purposes of research. Other participants were fearful that the research study would be unsuccessful in finding a cure and would leave them in poorer health than when they started.

*“For me to just up and change and do something different*, *I would have to have a lot of faith in what I was being presented*. *(…) I don't think I could take another body change*. *And my grandfather always told me*, *"If it ain't broke*, *don't fix it*.*" So*, *yeah*. *I just couldn't*. *It would feel like betrayal in a way*.*”*–Participant 002, Interview 2

*“Suppose it's not going to work*, *and now they've boosted your count and weakened your system*. *And their plan didn't work*, *so now you're just at a worse standpoint than [when] you started*. *(…) I definitely wouldn’t do [research]*.*”*–Participant 006, Interview 2

In additions to these health concerns, some participants would not participate due to barriers such as transportation or not knowing how to locate and enroll in research studies.

*“I don't know where to go to get into them*. *I don't have the information or whatever to get into those kind of researches*.*”*–Participant 003, Interview 1

“*Oh*, *now*, *transportation could be a problem*.*”*–Participant 001, Interview 1

#### Reasons to participate

Many of the reasons participants would agree to take part in HIV cure-related research were altruistic in nature. Reasons included wanting to help find a cure so they could be around for their children, improve the health of the transgender community and their partners, and build social cohesion between transgender women. A minority of participants would agree to participate because of hope that they would be cured of HIV, and even fewer participants were interested specifically in the monetary compensation.

*“I'll go and participate because I figure the more people that they can get information from*, *the better*, *especially in dealing with the transgender community*.*”*–Participant 002, Interview 1

*“If I can do something to help prolong somebody else's life*, *even though I might leave mine in the process*, *I would do it*.*”–*Participant 001, Interview 1

*“I'm going because I want to see a better outcome with this*. *You know what I mean*? *I want to see a better outcome*. *I don't want men like my boyfriend to fall prey to HIV or possibly catch AIDS*.*”*–Participant 002, Interview 2

*“So I think that a lot of transgender girls look for opportunities that would help us*. *You know what I mean*? *To be a little bit more independent*. *So that's why these studies that come along*, *a lot of girls do attend because it's how they stay connected in their own way*.*”*–Participant 002, Interview 1

#### Perceived benefits of HIV Cure-related studies

Perceived benefits of HIV cure-related studies were varied. Some believed studies would directly improve the participants’ health, while others recognized that participation was altruistic and would only benefit the health of others in the future. One participant cited the fame and fortune associated with being cured of HIV as a potential benefit of these studies.

*“The benefits would be better health*.*”*–Participant 009, Interview 2

*“The person actually participating is not benefiting*. *They're helping somebody else benefit off of what they get*.*”*–Participant 008, Interview 2

*“The fame*, *all that*. *Because you're probably famous*. *You're really going to be famous*, *and you really want to be rich*. *So there you go*.*”*–Participant 003, Interview 2

#### Perceived risks of HIV cure-related studies

Perceived risks of HIV cure-related studies included death, sickness, negative side effects, adverse reactions to experimental drugs, disclosure of participants’ HIV status, and not being able to successfully return to ART after the study is over.

*“Death*. *Sickness*. *(…) Other infections or reaction to it*. *Whatever it is*. *However they try and do it*. *(…) Being taken off your medicine and your virus mutating*. *Not being able to take that medicine you were taking*.*”*–Participant 009, Interview 2

*“The risk*, *I think*, *is that some people's bodies might reject their medication and they might end up dying from something that they're trying to help you to care for*. *I mean*, *I guess that's part of research*.*”–*Participant 001, Interview 2

*“I don't know if it can mess up what's going on inside your body or you got to worry about the side effects and all that other stuff*. *I mean*, *I'm all for it but I don't want to be the guinea pig*.*”*–Participant 007, Interview 2

When participants were asked specifically about their perceptions of side effects, they expressed concern about mental, physical, and emotional risks. Most participants could justify minor side effects which would subside after the study was over, but feared life-threatening problems such as heart conditions, seizures, and long-term health impacts. There was also concern for participating in a study for which the side effects were not yet known.

*“Yeah*, *the side effects*, *mainly the side effects because–and side effects cover a lot of things*, *mentally*, *physically*, *emotionally*.*”*–Participant 002, Interview 1

*“Like life-threatening side effects like fevers and heart problems and seizures and stuff like that*. *That would be scary*.*”–*Participant 009, Interview 1

*“I don't know*. *That's kind of hard to say*. *Because all of it sounds dangerous*. *And when it comes to the approach of something new*, *now that they came up with*, *that's trying to do something*, *because you don't know the side effects of it*.*”*–Participant 009, Interview 2

#### Perceptions of unacceptable risk in HIV cure-related studies

Unacceptable risks included some that signaled irrational fears. Others, however, included realistic concerns like discomfort or even death. Some participants would accept minor risks (e.g. headaches), but others considered those risks to be too high.

*“Let's hope I don't grow another eye on my forehead*.*”*–Participant 002, Interview 2

*“Anything besides death I'll deal with*. *I'll deal with a little headache and constipation and all that*, *but if it says death then I'm not dealing with this*.*”*–Participant 003, Interview 2

Another participant alluded to the frequency of depression among PLWHIV, highlighting the importance of monitoring psychosocial health throughout HIV cure-related studies.

*“Depression comes along with this*, *and depression makes you feel like everything is against you*. *Everything is against you*. *Like you're not going to win*. *You're at the finish line*, *you've had the training*, *you still didn't win*. *And that's depression*.*”–*Participant 006, Interview 2

Despite these concerns, one participant expressed needing to trust that researchers would ensure their safety throughout the study.

*“There's none*, *"too much of a risk"*. *Because*, *whatever's going on*, *they're going to make sure*, *if there's a vaccine or they're using needles*, *they're going to make sure I'm protected*.*”–*Participant 004, Interview 2

#### Concerns about HIV cure-related studies

Apart from direct side effects, concerns included participants’ uncertainty about the competency of researchers considering the irreversibility of certain modalities under investigation.

*“I ain't cool being a test subject*. *Meaning like*, *you shooting a vaccine into me*, *but do you know what it is that you're actually doing*? *Or what it is*? *Because once you put it in you can't just take it out as easy as you put it in*.*”–*Participant 008, Interview 2

An equal number of participants lacked such concerns and instead commented on the altruistic reasons for which one would agree to participate in HIV cure-related studies.

*“There are no concerns*. *None*, *because I'm helping someone–I'm helping myself*. *I'm helping […]*. *I'm helping […]*. *I'm helping […]*. *Whatever*, *whatever*. *It's to help people*. *We're put on this earth to help people*. *We're not supposed to be selfish*.*”*–Participant 004, Interview 2

#### Perceived burdens of HIV cure-related studies

While most concerns about participation in an HIV cure-related study included impacts on individuals’ physical and mental health, burdens associated with participating included confusion about what would be happening throughout the study and logistical factors such as the time required from participants and the need to travel to study sites.

*“Not knowing what's being done to you*.*”*–Participant 008, Interview 2

*“Time-consuming and—I'd say*, *basically*, *time-consuming*.*”*–Participant 009, Interview 2

*“The traveling*. *The traveling is really bad*. *Well*, *for certain people that's outside the area*.*”*–Participant 002, Interview 2

#### Potential impact of an HIV cure. *Personal impacts*

The majority of participants believed that the discovery of a cure for HIV would be life-changing. Anticipated impacts ranged from feeling liberated from a daily antiretroviral regimen and the fear of dying from HIV, to having the opportunity to be more selective when choosing future sexual partners or being afraid of having sex and acquiring HIV or other sexually-transmitted infections.

*“It would get better*. *I mean*, *I wouldn't have to worry about taking a pill every day now or any of that*.*”*–Participant 009, Interview 1

*“I would be careful of the people I picked as sexual partners*. *I would be careful on how I treat people*. *I would* [have] *so much gratefulness that I would have to get into something that was helping somebody else*. *That would push me just to make people's lives or things better*. *If there was a cure and I got the cure*, *and I was able to be cured from HIV*, *everything would change*.*”*–Participant 005, Interview 1

*“If I could get cured and got rid of this virus I would be scared to have sex*. *Because I wouldn't want to–I know what I've been through*. *I wouldn't want to have sex*. *I would want to have sex but I wouldn't have sex*.*”*–Participant 001, Interview 2

Conversely, a few participants felt that an HIV cure would have little impact on their lives because they would still have to take daily medication for comorbidities and/or practice safe sex to prevent the acquisition of other sexually transmitted infections. One participant expressed that she might refuse an HIV cure because, despite the associated challenges, living with HIV gives her strength.

*“I mean*, *it would make a difference*, *but I just wouldn't have to take HIV medications*, *but I would still have to take medications*, *me*, *myself because I have a heart condition*.*”*–Participant 001, Interview 1

*“Even if there was a so-called cure*, *I don't know if I would take it (…) because even though I have it*, *it's something about that struggle every day that gives me a little bit of strength*.*”*–Participant 002, Interview 1

#### Community impacts

When asked about the effects of an HIV cure on the transgender community, participants explained that they would no longer suffer from depression, may engage in less-risky behaviors, and may improve their lives in other ways by doing things like returning to school.

*“I think that it would be a good thing for the trans community*. *I think that a lot of people would wake up from the bitterness and the hurt*, *and the discrimination*, *and everything negative that they had to go through*, *it wouldn't matter no more*. *(…) So if somebody offered you the gift of the cure them things that bothered you before*, *it can't really bother you like that again because it's like you got a second chance at life*.*”*–Participant 005, Interview 1

Behavioral disinhibition was an emergent theme throughout interviews. Several participants hypothesized that individuals would engage in riskier sexual behaviors, knowing that if they acquired HIV, they could be cured.

*“Everybody would try to get it*, *and then they would think because oh*, *there's a cure*, *they're going to continue to have unprotected sex and don't worry about catching it again because they got a cure*.*”*–Participant 009, Interview 1

One participant questioned whether or not a cure would impact the transgender community at all with the concern that a cure would be reserved only for wealthy members of the population.

*“I think research got to be done to make a cure but once you accomplished the cure*, *then what*? *(…) Is you going to keep it*? *Is you going to try to figure out how can you (…) get money off it*? *Or is you just going to give it to people because they need it*?*”*–Participant 008, Interview 1

### Perceptions of HIV cure-related research strategies

#### Overall impression of HIV cure-related research strategies

Participants found HIV cure strategies to be interesting and asked many questions expressing their curiosity about the research.

*“It's magnificent and fascinating how advanced science is*, *wow*.*”–*Participant 004, Interview 2

*“Well*, *how long have they been doing these researches like this*? *(*…*) If they've been trying it this way for all these years*, *I mean maybe they need to try something else*. *Because if they've just been working at it since the '80s on this certain situation and no results yet*, *then they need to try to aim everything a different way*.*”*–Participant 003, Interview 2

*“When you go to dialysis*, *don't they flush your blood*? *They take impurities and stuff out your blood*. *So*, *why can't they do it that way*?*”*–Participant 006, Interview 2

While some participants were open to trying all of the strategies, others were apprehensive about trying any of them.

*“I would do all of them*. *(…) Because if one don't work*, *go to the next one*. *(…) I'm not afraid of basically trying anything when it comes to that*. *And it would help other people out*. *Because people helped me out*. *They get me where I'm at now*.*”*–Participant 001, Interview 2

*“So*, *truthfully*, *none of those would work for me because I'm focused on where I am right now*. *Unless I see a phenomenal turnaround*, *I'd just rather stay safe where I'm at*.*”*–Participant 002, Interview 2

#### Perceptions of latency reversing agents

A few participants were optimistic about latency reversing agents; however, the majority expressed concerns. In general, participants feared repercussions of reactivating HIV reservoirs.

*“What if it's a lot of them that's hiding [in HIV reservoirs]*? *What if they all decide to come out and then kill you*? *(…) That sounds like that person’s going to be real ill*. *They’re going to be real sick*.*”–*Participant 006, Interview 2

*“I wonder if the viruses–the bugs that you can't find but you wake them up*. *Will they start reproducing*?*”–*Participant 001, Interview 2

*“The part that keeps triggering my mind*, *when it wakes up*, *where it's going to be going*?*”–*Participant 007, Interview 2

*“It feels like I’m gambling at the casino with that one*. *(…) I come for an appointment*, *they say*, *‘Oh*, *well*. *We have to change it this time because it's constantly changing now*,*’ or*, *‘We found it*,*’ or*, *‘One was hiding somewhere*, *now it didn't spread (…) That's putting me on edge because I'm wondering*, *‘Is this going to work*? *Is this going to pull me through‴*–Participant 002, Interview 2

*“You don't really know what it's killing*, *when it's killing*. *Rather just say you're not only think it's killing something*, *then you wind up having brain damage or something like that*. *If the pill or whatever it is activates itself*, *then how can you stop it*?*”–*Participant 008, Interview 2

#### Perceptions of gene therapy and stem cell transplants

Several participants generally perceived gene modification and stem cell transplants to be risky and painful. Participants also expressed a lack of knowledge about somatic versus germline gene editing that impeded understanding the gene therapy approach.

*“Where is your DNA on the inside*? *Where*?*”*–Participant 007, Interview 2

*“Okay*. *That’s a little scary*.*”*–Participant 006, Interview 2

*“Wouldn't that be painful*? *Because that's like taking blood out and putting more blood in*.*”–*Participant 010, Interview 2

Interviewer: *“Okay*. *And then so you're saying the gene therapy seems the riskiest*?*”*

Participant: *“Yeah*. *Because it's messing with the genetic code*.*”–*Participant 009, Interview 2

*“So it's completely like going into a dark room*. *You don't know if it's in there right*.*”–*Participant 001, Interview 2

Nevertheless, after hearing the various strategies presented, gene editing was most commonly mentioned as the HIV cure-related research strategy participants would agree to participate in.

*“I think that I would probably go through with the putting a gene in my body*. *The stem cell*. *And then trying to boost my immune system up*. *I don't get sick much*, *but that would be a good thing*. *I wouldn't have to worry about catching any colds or anything*.*”–*Participant 001, Interview 2

*“Okay*. *The safest option might be number two* [gene therapy]. *(…) Because you can learn about the genes*, *you can know more about the people's body or whoever the patient is*. *That way you can work your way into knowing how to treat things*. *That seems to be easier and less complicated*.*”–*Participant 010, Interview 2

#### Perceptions of therapeutic vaccination strategies

Multiple participants utilized a military metaphor to make sense of therapeutic vaccination or immune-based strategies.

*“That’s just like a shield*, *right*? *(…) Okay*, *it sounds crazy*, *but it's like Truvada with a sword (…) It sounds like a raging war inside my body*.*”*–Participant 006, Interview 2

Several participants were concerned that the vaccine would cause illness and cited previous experiences in which they or someone they knew became ill after receiving a vaccination.

*“I would be scared of the vaccinations*. *(…) Because I mean*, *you’d get so sick*. *(…) I think that is very dangerous*.*”–*Participant 001, Interview 2

*“You take for instance*, *when the H1N1 flu and all that came out*, *I never got the flu shot*. *Never took the H1N1*. *So one year I'm just like*, *‘Okay*. *I'm going to take it*.*’ The worst thing I ever did*. *I was sick for three weeks*. *I said*, *‘I'm never taking that shot again*.*’ So that would be my support and evidence*. *I went through this before*.*”*–Participant 008, Interview 2

Other participants were apprehensive about this approach because they understood that HIV can mutate, and they were concerned that all strains of HIV may not be prevented with a single vaccine.

*“Does the infection fight back*? *Don’t it fight back*? *That’s when it mutates and stuff*. *(…) So suppose they shoot you with the shot*, *and the strain that you carry is stronger than—and what it if it takes you down*? *(…) That’s just my concern with the vaccine*.*”*–Participant 006, Interview 2

*“By there being so many different strands*, *would there be different vaccines you can get or would there just be one that they're trying to make that work*? *For all the different strands of HIV*? *(…) I would say that would be kind of hard*, *really*.*”*–Participant 001, Interview 2

One participant in particular expressed distrust toward researchers and a lack of confidence in their competency to formulate a vaccine which would be effective and safe.

*“The [vaccine approach] is just a lie because a vaccine whether it's a pill*, *whether it's a egg shot or whatever*, *they shoot you with something*. *So*.*”*- Participant 008, Interview 2

As a result of all the above concerns, most participants said vaccines seemed the riskiest HIV cure-related research strategy.

### Part 2: Perceptions of HIV treatment and treatment Interruptions

#### Perceived advantages of HIV medications

Perceived benefits of HIV medications included improved appetite, energy levels, and mental health; prolonged life; and hope for the future. Participants also commented on the positive attributes of their current ART, such as having no adverse side effects, being able to take a single tablet regimen, and small pill sizes.

*“The medicine has really gave [sic] me an opportunity to live*. *You know what I mean*? *I've gained weight*. *It helped me to eat*. *I even get a little rest*. *You know what I mean*? *It's really changed my life*. *I don't have the depression that I used to have*.*”–*Participant 002, Interview 2

*“Well*, *nothing's bad about it because I don't get side effects from it*. *And what's good about it is that I feel normally healthy*, *and I'm not sick*, *and I'm undetectable and it just keeps me healthy*.*”*–Participant 009, Interview 2

*“The good thing to me about it is that I have to just take one little tiny pill*.*”*–Participant 007, Interview 2

#### Perceived drawbacks of HIV medications

While some participants had only positive things to say about current HIV medications, others described drawbacks to their treatment regimens. Some reported experiencing unpleasant side effects such as bloating, drowsiness, and stomach aches. A few participants commented on the toll that taking a daily pill for HIV can have on one’s mental health.

*“Once you do something long enough*, *it becomes natural*. *But it really shouldn't be a natural thing of life to get up every day and take a pill to survive*. *That's not how life's supposed to be*.*”*–Participant 002, Interview 2

*“It's a constant reminder*. *A constant reminder*. *I try to just chuck a handful of my pills*. *I take hormone pills*, *and I take testosterone blockers*. *So I just chuck them all*, *yeah*.*”*–Participant 006, Part 2

#### Willingness to interrupt HIV treatment

Almost all participants were willing to participate in treatment interruptions; however, several had certain conditions such as receiving monetary compensation, being closely monitored, and interrupting ART for a very limited time. One participant felt comfortable knowing that treatment could be resumed at any time.

*“I don't think it would be bad because I would be monitored*. *If things start going bad*, *then they would probably know what to do*, *I hope*.*”*–Participant 010, Interview 1

*“It depends on how long I would have to stop*. *I don't know*, *gosh*. *The longest time I would stop would be for a month*.*”*–Participant 001, Interview 1

*“I would do it for a study*. *Yeah because like I said I can always get back on*.*”–*Participant 003, Interview 1

#### Concerns about HIV treatment interruptions

Some participants were hesitant to participate in an HIV cure-related study requiring treatment interruption. A common theme was fear that HIV could mutate during the treatment interruption, rendering their antiretroviral regimen ineffective once the interruption ended.

*“And if there’s any change in—with the medicine that I’m taking*, *will I be able to go back to my medicine again*.*”*–Participant 002, Interview 1

*“They don’t really have that many medicines out here*. *It’s hard to find medicine that your body’s going to work with if you keep messing up all the medicine that they give you*, *and some have ran out of options*.*”*–Participant 009, Interview 1

*“I would be very fearful of stopping my HIV medicine because I know how fast the virus can grow*. *(…) How long would I have to stop it*? *And if I do stop it [for] whatever research you're all doing to find this cure*, *what are these drugs going to do to me*? *Are they going to make me sick*?*”*–Participant 005, Interview 1

### Part 3: Considerations for transgender women and HIV cure-related research

#### Competing priorities for transgender women

Participants expressed many other priorities than HIV cure-related research. These competing priorities included living a normal life by having a home, job, and family; receiving non-discriminatory health care; being perceived and accepted as a woman; having romantic relationships; achieving financial stability; accessing gender-affirming hormones; remaining safe and avoiding violence; receiving respect and not being stigmatized for their gender identity; and maintaining positive mental and behavioral health.

Only one participant identified finding a cure for HIV as her top priority.

*“Now that that cure word is mentioned*, *that would be number one*. *That would be the first thing on the list*.*”*–Participant 005, Interview 1

Some participants did not prioritize HIV cure-related research because they had access to HIV treatment which enables them to live longer and healthier lives. Others noted that discovering a cure would not significantly alter their day-to-day lives or change their behaviors.

*“It’s not a priority*, *no*. *(…) Because people can live with HIV and live their full lifespan and die from natural causes and not from the disease*.*”*–Participant 009, Interview 1

*“Right now*, *I really don't care because I'm still going to be cautious*. *I'm still going to have safe sex when I do decide to have it*. *And it's not going to make me run out and say*, *hey*, *boys I'm free*. *No*. *That's not going to work*.*”*–Participant 010, Interview 1 & 2

#### Necessary protections for HIV cure-related studies

Participants outlined specific protections they would expect if they decided to participate in an HIV cure-related study. They would want to be informed up front about any risks and side effects they may experience and would want the autonomy to make the decision to participate without coercion. Ideally, participants would like to hear from someone who was previously a participant to learn about their experiences with the study.

*“Be honest*. *It won't take much*. *You know what I mean*? *If you're doing something and there's a choice*, *I think that it's very important to always make sure that a person know that they have a choice*. *(…) The ethical thing to do is to always make sure that person has a voice and that you're being honest*. *(…) Because if something bad just starts happening and you didn't tell me about it*, *but you knew it was going to happen*, *there is no ethics in that*.*”*–Participant 002, Interview 2

*“Have someone that's gone through it before you that could give you their experience (…) I think that would be another way to sooth the mind of someone that doesn't know what to expect*.*”*–Participant 005, Interview 1

Those who decide to participate would expect their health to be monitored through regular check-ups and to be provided with medical assistance should something go wrong. Participants would want to be kept informed about their health throughout the study but would expect that information be kept confidential. Should participants decide they no longer want to be in the study, they would expect to be able to return to their previous antiretroviral regimen.

*“I would really want to know that they had my back*. *If something went wrong*, *are you going to make sure that people know that I'm doing this*, *or I was in this situation*? *(…) Are you willing to make sure I'm okay because I decided to try something that was supposed to benefit everybody else*?*”–*Participant 002, Interview 1

*“Paid compensation*. *Extra healthcare*. *24/7 awareness*. *When I say that*, *I mean*, *constant checkup*. *Okay*? *Constant checkup*. *And back to the privacy*. *Confidential*. *As long as it's confidential*.*”*–Participant 006, Interview 1

Participants expressed a strong desire to be treated with respect and to know that the strategy being offered to them had true potential to be effective.

*“You have someone's life in your hand*. *You're controlling someone else's health*. *(…) You have to worry about that person who you are experimenting on*. *And that's what I think most researchers should really consider*. *I'm just not a cadaver or–I'm a living person*.*”*–Participant 001, Interview 2

“*Have evidence that what they're trying to do has a possibility of it working (…) Evidence*. *Okay*? *Not*, *‘I'll take it and try this one*, *and then try this one*, *and try this one*.*’ Nope*. *(…) I don't want to go through that*.*”*–Participant 006, Interview 2

#### Considerations specific for transgender women

A common priority among participants was that they be accepted as woman and treated just like everyone else who may participate in research.

*“I've been to an interview before*, *and they were using ‘He’ (…) that it was really uncomfortable because it was a man*. *And that was like–I was like*, *‘I just can't do this one*.*’ He referred to us as*, *‘He*.*’”*–Participant 002, Interview 1

*“It's just we're all participating as humans*, *not as different genders or races or anything*, *just as a person that's trying to help find a cure*. *(…) It shouldn't be like trans-women should be singled out*. *We should be just a candidate just like anyone else*.*”*–Participant 003, Interview 1

Participants wanted researchers to be sensitive to the diversity among transgender women, avoid being judgmental, and respect that some questions may be too personal to ask.

*“Their personalities*. *Their lifestyle*. *(…) All of them got different personalities*. *And*, *it's just like meeting different people*. *We all take information differently*. *And some of them feel like*, *"Oh*. *It's too personal*. *This and that*.*" And lifestyle is like acceptance of who they are and not having them feel judged*, *us feel judged*. *That's it*.*”*–Participant 009, Interview 2

#### Information for transgender women

Participants expressed a desire to receive as much information as possible about the research. Beforehand, participants would like to know what research is being done and why, potential risks and side effects, and anything else about the study which may help participants understand what they are volunteering to do.

*“All that's available*. *Everything that they know*, *we should know*. *Not how it's done*, *but why it's done and when it's done and the cause and side effects and all*, *everything*. *All the information*.*”*–Participant 009, Interview 2

Following the study, participants would appreciate hearing about the findings from the study and anything that may have been discovered as a result of their participation. Suggested modalities to deliver various types of information included newsletters and YouTube videos.

*“I think they should get information of how it's coming along with the study*. *I participated in so many studies*, *but I never know where they go from there*. *You know what I mean*? *(…) once you've got up enough data that you can figure–at least let us know what's going on*.*”*–Participant 002, Interview 2

#### Strategies to encourage participation of transgender women in HIV cure-related research

Participants often referred to the sense of community which exists in the transgender, lesbian, gay, and bisexual community population, particularly in the Baltimore area. This network should be utilized to share information about HIV cure-related studies.

*“Go into these streets and these areas where they be lingering and being at and social events that they be having out in the cities like Pride*. *And our state*, *but all these other little festivals*. *And handing out flyers or posting things on social media*, *where a lot of people be at*.*”*–Participant 009, Interview 2

A desire to create an even greater sense of community among HIV cure-related research transgender participants was an emergent theme from the interviews. Following recruitment, participants requested that studies establish a sense of community among those participating to strengthen those connections.

*“Just because you call us in to say*, *"Oh*, *come get this $50 and do this thing*,*" that doesn't help us communicate with each other or know what's going on within our community*, *(…) So if y'all are so interested in making sure that we're good*, *pull us together and let's have a community powwow of a transgender meeting*. *And y'all can let us know where you're coming from*, *and we can let you know where we're coming from*.*”*–Participant 002, Interview 2

*“I don't think that it should be just for trans*. *I think that it should be for the LGBT–the whole community (…) but it needs to be in a group setting (…) with different settings of people*.*”–*Participant 006, Interview 2

Participants requested that researchers strongly consider their safety by providing transportation or other means to safely travel to and from the study locations.

*“Except*, *especially in the evening*, *most studies need to have little cab vouchers or some tokens for the people*. *Because when you give them the card*, *sometimes when you give them the cash*, *you can't just go out and get change*. *Not anymore*. *It's not safe*. *So it's taking all of that into consideration*.*”*–Participant 010, Interviews 1 & 2

## Discussion

This qualitative study explored Black transgender women’s perceptions of HIV cure-related research and attitudes toward participation; perceptions of HIV treatment and interruptions; and considerations for engaging Black transgender women in HIV cure-related research. Salient themes included skepticism about HIV cure strategies and limited benefits compared with an undetectable viral load. Willingness to interrupt treatment for research was low and was linked to being able to go back on the same HIV treatment without consequence when the study ended. Concerns about being a test subject and perceptions of risks versus benefits of various strategies also affected willingness to take part in HIV cure-related research. Centering the dignity and autonomy of research participants as well as building on and supporting existing social networks were identified as important for engaging Black transgender women in HIV cure-related research. Many of the themes identified in this study echo findings from research with other key populations [16, 24].

Implicit distrust of research emerged as an overarching theme across many narratives. Participants questioned whether researchers would fully disclose risks, take care of them if something went wrong, or share the benefits of a cure equitably. This theme is consistent with prior research identifying medical distrust as a barrier to Black people’s engagement with HIV prevention and care [25–28]. In a recent qualitative study, Black women explained how a history of mistreatment within medical settings and ineffective, sometimes inaccurate, communication from healthcare providers led to mistrust[28]. Another study with focus group participants living with HIV found distrust in research to be an emergent theme[24]. In particular, the belief that any cure would be reserved for the wealthy resonating with that of several participants in this study.

Participants in this study only considered HIV cure to be a complete elimination of HIV. Perceived benefits of HIV cure included freedom from taking medication and from worries about HIV transmission to sexual partners. These findings were similar to results from Sylla et al.’s study involving 10 focus groups with 76 people living with HIV across four U.S cities [16]. However, unlike the prior study, none of the Black transgender women in this study raised de-stigmatization as a potential benefit of HIV cure. It is possible that Black transgender women did not raise this issue because they understand that they would still continue to experience stigma based on their gender and race [29–32]. Or they may not have raised stigma because they experience having an undetectable (therefore untransmittable [33]) viral load to be as destigmatizing as an HIV cure.

Similar to other qualitative research [34], we found that most participants were satisfied with their current antiretroviral regimens and some saw limited benefit to an HIV cure or remission over ART. Given the acceptability of ART, it was not surprising that most participants expressed reluctance to interrupt therapy to help advance HIV cure-related research. Similar to other socio-behavioral studies around HIV cure-related research among PLWHIV, viral rebound was a major concern for participants [35–37]. These findings are consistent with conclusions from a previous qualitative study[37] in which PLWHIV, clinician-researchers, and bioethicists described the care needed in determining when and how to deploy treatment interruptions, including the need for intensive and frequent monitoring, a clear back-up ART option if resistance develops, criteria for reinstituting ART, and provision of adequate and clear information for study participants.

Participants commonly reported altruism as a reason for participating in HIV cure-related research, similar to participants in other studies [15, 19, 35, 38]. Several transgender women specifically noted the desire to benefit other transgender women and expand the evidence base for their community. Concerns about being treated like a guinea pig echo those raised in other research on attitudes toward HIV cure-related research [24]. In addition to concerns about the potential sequelae of HIV treatment interruptions, participants were also concerned about the effects of specific strategies on their health. However, unlike previous studies [15], several participants stated there were no risks too great to take–suggesting that either participants were not fully aware of the potential risks or, less likely given the context of their other statements, they were willing to risk everything for an HIV cure.[39] Findings may also underscore the need to communicate trial-related risks and safeguards in place to minimize risks to study participants using language and a format that are community-friendly.

Very little research has explored perceptions of *specific* HIV cure-related research strategies. Dubé et al. conducted one qualitative study on perceptions of cell and gene therapy with Black cisgender participants in the US Northwest [24]. In that study, participants were quite concerned about the risk associated with the approach, voiced significant mistrust of research in general, and believed there was already a cure from HIV that was being withheld from the poor. While some Black transgender women in this study also expressed a general mistrust of research, few felt a cure already existed. However, they did note that when one was found, it would be reserved for the wealthy.

Our study was unique in asking about reactions to a number of different HIV cure-related research strategies–latency reversing agents, gene modification and stem cell transplants, and therapeutic vaccines. Interestingly, most participants voiced significant concern about therapeutic vaccines, compared with the other strategies. Many reported negative experiences with other vaccines and were reluctant to participate in a study using this approach. Overall, participants had many questions about each of the strategies and many did not have the educational background or medical literacy that would prepare them to understand these approaches and their rationale. This further suggests the need for broad education for transgender women on HIV pathogenesis as well as HIV cure-related research. As researchers seek to engage Black transgender women in research, it will be important to consider their completing priorities such as housing, financial security, gender affirmation, hormone therapy, and safety from endemic anti-transgender violence.

Many concerns raised by Black transgender women mirror those raised by other populations, including satisfaction with current single-tablet ART regimens, reluctance to interrupt treatment for research, and concerns about specific HIV cure-related research approaches. However, concerns specific to transgender women included the desire for research staff to be gender-affirming and to promote community among transgender women as well as address safety issues associated with risk of exposure to transphobic violence when traveling to study visits. In addition to addressing these issues, participants stressed the importance of HIV cure-related researchers providing accessible, complete information and expressing genuine care and concern for transgender communities.

This study had several limitations. It was conducted at a single research site in Baltimore with a limited number of study participants, and results may not be transferable to transgender women living with HIV in other settings. Study participants had limited prior knowledge about HIV cure-related research; however, the interviewer mitigated this challenge by providing community-friendly information in lay terms. Finally, selection bias may have led to a sample of participants with generally favorable views about HIV research. These limitations notwithstanding, we were able to conduct two in-depth interviews with nine of ten study participants and build rapport with all the participants. Our findings represent a breadth of views from transgender women who have been nearly invisible in HIV cure-related research.

Black transgender women are disproportionately affected by HIV and would benefit from an effective HIV cure. Engaging Black transgender women in HIV cure-related research will require attention to the specific concerns voiced by this community. Inclusion is both a matter of justice and a necessity for good science [40–42]. Table 3 provides recommendations for engagement of Black transgender women in HIV cure-related research based on these study findings. Failure to recruit or identify transgender participants leaves critical biological and social questions unanswered about not only if, but why and how various HIV cure strategies may work for all people living with HIV.

**Table 3:** Recommendations for engaging black transgender women in HIV cure-related research.

Recommendation 1	Complete the five modules of the Transgender Training Curriculum for HIV Research developed by the NIH DAIDS Cross-Network Transgender Working Group. All trainings are available as e-learning modules and in-person training tools. Modules can be accessed via the DAIDS Learning Portal: https://daidslearningportal.niaid.nih.gov.
Recommendation 2	Build HIV cure-related research protocols with gender-affirming and racial equity framework using a trauma-informed research lens using knowledge and skills.
Recommendation 3	Train and/or mentor researchers from the Black transgender community and hire Black transgender women onto the research staff.
Recommendation 4	Ensure transgender participants feel welcome at research sites and are referred for research opportunities; research teams should provide information on how HIV cure-related research protocols affect the transgender community; establish academic-community partnerships with organizations that have experience engaging with the transgender community, include Black transgender-led organizations.
Recommendation 5	Initiate and maintain dialogue about HIV cure-related research with the Black transgender community, for example, informational community forums.

## Supporting information

S1 Appendix. Interview guides (DOCX)

S1 Table. Supplemental quotes (DOCX)

## References

[1] Becasen JS, Denard CL, Mullins MM, Higa DH, Sipe TA. Estimating the Prevalence of HIV and Sexual Behaviors Among the US Transgender Population: A Systematic Review and Meta-Analysis, 2006–2017. Am J Public Health. 2018:e1–e8. Epub 2018/11/30. 10.2105/AJPH.2018.304727 . 30496000 PMC6301428

[2] Poteat T, Hanna DB, Rebeiro PF, Klein M, Silverberg MJ, Eron JJ, et al Characterizing the Human Immunodeficiency Virus Care Continuum Among Transgender Women and Cisgender Women and Men in Clinical Care: A Retrospective Time-series Analysis. Clinical Infectious Diseases. 2019 10.1093/cid/ciz322 31573601 PMC7319059

[3] Bukowski LA, Chandler CJ, Creasy SL, Matthews DD, Friedman MR, Stall RD. Characterizing the HIV Care Continuum and Identifying Barriers and Facilitators to HIV Diagnosis and Viral Suppression Among Black Transgender Women in the United States. Journal of acquired immune deficiency syndromes (1999). 2018;79(4):413–20. 10.1097/QAI.0000000000001831 . 30080750 PMC6521857

[4] Rosen JG, Malik M, Cooney EE, Wirtz AL, Yamanis T, Lujan M, et al Antiretroviral Treatment Interruptions Among Black and Latina Transgender Women Living with HIV: Characterizing Co-occurring, Multilevel Factors Using the Gender Affirmation Framework. AIDS Behav. 2019;23(9):2588–99. Epub 2019/07/03. 10.1007/s10461-019-02581-x 31263998 PMC6768710

[5] Teti M, Bauerband LA, Altman C. Adherence to Antiretroviral Therapy Among Transgender and Gender Nonconforming People Living with HIV: Findings from the 2015 U.S. Trans Survey. Transgender health. 2019;4(1):262–9. 10.1089/trgh.2019.0050 . 31656854 PMC6814081

[6] Curno MJ, Rossi S, Hodges-Mameletzis I, Johnston R, Price MA, Heidari S. A Systematic Review of the Inclusion (or Exclusion) of Women in HIV Research: From Clinical Studies of Antiretrovirals and Vaccines to Cure Strategies. Journal of acquired immune deficiency syndromes (1999). 2016;71(2):181–8. Epub 2015/09/12. 10.1097/QAI.0000000000000842 . 26361171

[7] Jilg N, Li JZ. On the Road to a HIV Cure: Moving Beyond Berlin and London. Infect Dis Clin North Am. 2019;33(3):857–68. 10.1016/j.idc.2019.04.007 . 31395147 PMC6814144

[8] Gupta RK, Abdul-Jawad S, McCoy LE, Mok HP, Peppa D, Salgado M, et al HIV-1 remission following CCR5Delta32/Delta32 haematopoietic stem-cell transplantation. Nature. 2019;568(7751):244–8. Epub 2019/03/06. 10.1038/s41586-019-1027-4 . 30836379 PMC7275870

[9] Kuo L, Lawrence D, McDonald D, Refsland E, Bridges S, Smiley S, et al Highlights from the Fourth Biennial Strategies for an HIV Cure Meeting, 10–12 October 2018, Bethesda, MD, USA. J Virus Erad. 2019;5(1):50–9. . 30800428 10.1016/S2055-6640(20)30280-6 PMC6362907

[10] New Report: Global Investment in HIV Cure Research and Development. September 23, 2020.: AVAC; [November 1, 2020]. Available from: https://www.avac.org/blog/new-report-global-investment-hiv-cure-research-and-development.

[11] Blankson JN, Siliciano JD, Siliciano RF. Finding a cure for human immunodeficiency virus-1 infection. Infect Dis Clin North Am. 2014;28(4):633–50. Epub 2014/09/30. 10.1016/j.idc.2014.08.007 . 25277513 PMC4253590

[12] Dresser R. First-in-human HIV-remission studies: reducing and justifying risk. J Med Ethics. 2017;43(2):78–81. Epub 2016/05/05. 10.1136/medethics-2015-103115 . 27143494

[13] Dubé K, Henderson GE, Margolis DM. Framing expectations in early HIV cure research. Trends Microbiol. 2014;22(10):547–9. Epub 2014/10/05. 10.1016/j.tim.2014.08.003 25280965 PMC4201845

[14] Julg B, Dee L, Ananworanich J, Barouch DH, Bar K, Caskey M, et al Recommendations for analytical antiretroviral treatment interruptions in HIV research trials-report of a consensus meeting. Lancet HIV. 2019;6(4):e259–e68. Epub 2019/03/20. 10.1016/S2352-3018(19)30052-9 30885693 PMC6688772

[15] Dubé K, Taylor J, Sylla L, Evans D, Dee L, Burton A, et al 'Well, It's the Risk of the Unknown… Right?': A Qualitative Study of Perceived Risks and Benefits of HIV Cure Research in the United States. PloS one. 2017;12(1):e0170112-e 10.1371/journal.pone.0170112 . 28122027 PMC5266311

[16] Sylla L, Evans D, Taylor J, Gilbertson A, Palm D, Auerbach JD, et al If We Build It, Will They Come? Perceptions of HIV Cure-Related Research by People Living with HIV in Four U.S. Cities: A Qualitative Focus Group Study. AIDS Res Hum Retroviruses. 2018;34(1):56–66. 10.1089/AID.2017.0178 . 29198134 PMC5771509

[17] Dubé K, Hosey L, Starr K, Barr L, Evans D, Hoffman E, et al Participant Perspectives in an HIV Cure-Related Trial Conducted Exclusively in Women in the United States: Results from AIDS Clinical Trials Group 5366. AIDS Res Hum Retroviruses. 2020;36(4):268–82. Epub 2020/03/13. 10.1089/AID.2019.0284 32160755 PMC7185351

[18] Grossman CI, Ross AL, Auerbach JD, Ananworanich J, Dubé K, Tucker JD, et al Towards Multidisciplinary HIV-Cure Research: Integrating Social Science with Biomedical Research. Trends Microbiol. 2016;24(1):5–11. Epub 2015/12/09. 10.1016/j.tim.2015.10.011 26642901 PMC4698010

[19] Dubé K, Evans D, Sylla L, Taylor J, Weiner BJ, Skinner A, et al Willingness to participate and take risks in HIV cure research: survey results from 400 people living with HIV in the US. J Virus Erad. 2017;3(1):40–50.e21. . 28275457 10.1016/S2055-6640(20)30295-8 PMC5337420

[20] Reisner SL, Chaudhry A, Cooney E, Garrison-Desany H, Juarez-Chavez E, Wirtz AL. 'It all dials back to safety': A qualitative study of social and economic vulnerabilities among transgender women participating in HIV research in the USA. BMJ Open. 2020;10(1):e029852 Epub 2020/01/22. 10.1136/bmjopen-2019-029852 31959600 PMC7044992

[21] The Well Project. Finding a Cure for HIV 2019 [January 21, 2020]. Available from: http://www.thewellproject.org/hiv-information/finding-cure-hiv#Current_cure_research_strategies.

[22] Hsieh HF, Shannon SE. Three approaches to qualitative content analysis. Qual Health Res. 2005;15(9):1277–88. Epub 2005/10/06. 10.1177/1049732305276687 . 16204405

[23] Raskind IG, Shelton RC, Comeau DL, Cooper HLF, Griffith DM, Kegler MC. A Review of Qualitative Data Analysis Practices in Health Education and Health Behavior Research. Health Educ Behav. 2019;46(1):32–9. Epub 2018/09/19. 10.1177/1090198118795019 30227078 PMC6386595

[24] Dubé K, Simoni J, Louella M, Sylla L, Mohamed ZH, Patel H, et al Acceptability of Cell and Gene Therapy for Curing HIV Infection Among People Living with HIV in the Northwestern United States: A Qualitative Study. AIDS Res Hum Retroviruses. 2019;35(7):649–59. Epub 2019/05/21. 10.1089/AID.2019.0021 . 30990052 PMC6602097

[25] Bogart LM, Ransome Y, Allen W, Higgins-Biddle M, Ojikutu BO. HIV-Related Medical Mistrust, HIV Testing, and HIV Risk in the National Survey on HIV in the Black Community. Behav Med. 2019;45(2):134–42. Epub 2019/07/26. 10.1080/08964289.2019.1585324 31343966 PMC6783255

[26] Pellowski JA, Price DM, Allen AM, Eaton LA, Kalichman SC. The differences between medical trust and mistrust and their respective influences on medication beliefs and ART adherence among African-Americans living with HIV. Psychol Health. 2017;32(9):1127–39. Epub 2017/05/06. 10.1080/08870446.2017.1324969 . 28475365 PMC8034835

[27] Zhang C, McMahon J, Leblanc N, Braksmajer A, Crean HF, Alcena-Stiner D. Association of Medical Mistrust and Poor Communication with HIV-Related Health Outcomes and Psychosocial Wellbeing Among Heterosexual Men Living with HIV. AIDS Patient Care STDS. 2020;34(1):27–37. Epub 2019/11/23. 10.1089/apc.2019.0200 31755736 PMC6983731

[28] Randolph SD, Golin C, Welgus H, Lightfoot AF, Harding CJ, Riggins LF. How Perceived Structural Racism and Discrimination and Medical Mistrust in the Health System Influences Participation in HIV Health Services for Black Women Living in the United States South: A Qualitative, Descriptive Study. J Assoc Nurses AIDS Care. 2020;31(5):598–605. Epub 2020/09/02. 10.1097/JNC.0000000000000189 . 32868634

[29] Goldenberg T, Jadwin-Cakmak L, Popoff E, Reisner SL, Campbell BA, Harper GW. Stigma, Gender Affirmation, and Primary Healthcare Use Among Black Transgender Youth. Journal of Adolescent Health. 2019 10.1016/j.jadohealth.2019.04.029. PMC7296990 31303554

[30] Brooks RA, Cabral A, Nieto O, Fehrenbacher A, Landrian A. Experiences of Pre-Exposure Prophylaxis Stigma, Social Support, and Information Dissemination Among Black and Latina Transgender Women Who Are Using Pre-Exposure Prophylaxis. Transgender health. 2019;4(1):188–96. 10.1089/trgh.2019.0014 . 31482134 PMC6716188

[31] White Hughto JM, Reisner SL, Pachankis JE. Transgender stigma and health: A critical review of stigma determinants, mechanisms, and interventions. Social science & medicine (1982). 2015;147:222–31. Epub 2015/11/11. 10.1016/j.socscimed.2015.11.010 . 26599625 PMC4689648

[32] Poteat T, German D, Kerrigan D. Managing uncertainty: a grounded theory of stigma in transgender health care encounters. Soc Sci Med. 2013;84:22–9. Epub 2013/03/23. 10.1016/j.socscimed.2013.02.019 . 23517700

[33] Prevention Access Campaign [May 10, 2020]. Available from: https://www.preventionaccess.org/.

[34] Ma Q, Wu F, Henderson G, Rennie S, Rich ZC, Cheng Y, et al 'I can coexist with HIV': a qualitative study of perceptions of HIV cure among people living with HIV in Guangzhou, China. J Virus Erad. 2016;2(3):170–4. . 27482457 10.1016/S2055-6640(20)30465-9 PMC4967969

[35] Power J, Westle A, Dowsett GW, Lucke J, Tucker JD, Sugarman J, et al Perceptions of HIV cure research among people living with HIV in Australia. PloS one. 2018;13(8):e0202647-e 10.1371/journal.pone.0202647 . 30142171 PMC6108463

[36] Lau JSY, Smith MZ, Allan B, Martinez C, Power J, Lewin SR, et al Perspectives on Analytical Treatment Interruptions in People Living with HIV and Their Health Care Providers in the Landscape of HIV Cure-Focused Studies. AIDS Res Hum Retroviruses. 2019:10.1089/AID.2019.0118. 10.1089/AID.2019.0118 . 31608648 PMC7185349

[37] Dubé K, Evans D, Dee L, Sylla L, Taylor J, Skinner A, et al "We Need to Deploy Them Very Thoughtfully and Carefully": Perceptions of Analytical Treatment Interruptions in HIV Cure Research in the United States-A Qualitative Inquiry. AIDS Res Hum Retroviruses. 2018;34(1):67–79. Epub 2017/07/10. 10.1089/AID.2017.0067 . 28562069 PMC5771546

[38] Gilbertson A, Kelly EP, Rennie S, Henderson G, Kuruc J, Tucker JD. Indirect Benefits in HIV Cure Clinical Research: A Qualitative Analysis. AIDS Res Hum Retroviruses. 2019;35(1):100–7. Epub 2018/08/22. 10.1089/AID.2017.0224 . 30009625 PMC6343180

[39] Dubé K, Dee L. Willingness to risk death endpoint in HIV cure-related research with otherwise healthy volunteers is misleading. J Virus Erad. 2020;6(2):81–4. . 32405426 10.1016/S2055-6640(20)30021-2 PMC7213068

[40] Dubé K, Sylla L, Dee L, Taylor J, Evans D, Bruton CD, et al Research on HIV cure: Mapping the ethics landscape. PLoS Med. 2017;14(12):e1002470-e 10.1371/journal.pmed.1002470 . 29220353 PMC5722280

[41] Scully EP. Sex Differences in HIV Infection. Curr HIV/AIDS Rep. 2018;15(2):136–46. 10.1007/s11904-018-0383-2 . 29504062 PMC5882769

[42] Gianella S, Tsibris A, Barr L, Godfrey C. Barriers to a cure for HIV in women. Journal of the International AIDS Society. 2016;19(1):20706–. 10.7448/IAS.19.1.20706 . 26900031 PMC4761692

