# Supplementary material for: “I would really want to know that they had my back”: Transgender women’s perceptions of HIV cure-related research in the United States
Source: PLoS One. 2020 Dec 31;15(12):e0244490. doi: 10.1371/journal.pone.0244490 (PMC7774946; doi:10.1371/journal.pone.0244490)
Supplement: S1 Appendix. Interview guides — (DOCX) [file pone.0244490.s001.docx]

**S1. Appendix Interview Guides**

**INTERVIEW ONE**

**Introduction**

1. Let’s start with talking about research.
   1. What comes to mind when you think of research?
   2. Can you tell me about your history of participating in research?
      1. If yes, what kind of research was it? [*Probe to see if it was for trans women explicitly*]
         1. What was it like to participate in this research?
      2. If no, why haven’t you participated in research?
      3. What appeals to you about participating in research?
      4. Is there anything you do not like about participating in research studies? If yes, please tell me more about that.
      5. Some people avoid health research because they don’t trust the healthcare system. What are you feelings about the healthcare system?
         1. When you hear the word “trust”, what does that mean to you?

**Perceptions of HIV**

1. What is your understanding of what causes HIV/AIDS?
   1. What you have heard about causes of HIV?
   2. What do you believe about causes of HIV?
2. Some people have told us that they believe that HIV/ AIDS is man-made. Others have said that there is a cure but it is being withheld. We have also come across people who think that HIV was created to infect particular groups. Have you ever heard of these ideas or any ideas similar to these? What do you think about those ideas?

**Perceptions of and Experience with HIV Cure Research**

1. Can you tell me about your history of participating in HIV research?
   1. If yes, what kind of research was it? [*Probe to see if it was for trans women explicitly*]
   2. What was it like to participate in this research?
   3. What appeals to you about these studies?
   4. Is there anything you do not like about these studies? If yes, please tell me more about that.
2. What comes to mind when you hear the words “HIV cure”?
3. What do you think transgender women know about HIV cure research?
4. Have you participated in any HIV cure research?
   1. If yes, probe for details.
   2. If no, ask reasons why not.
5. What do you think would change if researchers found a cure for HIV?
   1. How do you think this would impact you?
   2. How do you think this would impact the transgender community?

**Barriers and Facilitators**

1. What do you imagine would be involved in joining an HIV cure study?
2. Why do you think a person might want to join an HIV cure study?
   1. Are there things about an HIV cure study that might make YOU more willing to participate?
3. Why do you think a person might NOT want to join an HIV cure study?
   1. Are there things about an HIV cure study that might make YOU less willing to participate?
4. Do you think there are different things a transgender woman should think about before joining a cure study compared with women who aren’t transgender? Compared with men?
5. If you wanted to join an HIV cure study, what are the kinds of things that would make it hard for you to do it?
   1. Are there specific things that researchers can and should do to make it easier for you to participate?
6. In order for someone living with HIV to participate in ***some*** of the HIV cure studies, they would have to stop taking their HIV treatment. What do you think would motivate someone doing well on HIV treatment to participate in an HIV cure study?
   1. Do you think you would you be willing to stop taking your HIV treatment for some period of time in order to participate in an HIV cure study?
   2. What concerns do you have about stopping HIV treatment for some period of time in order to participate in an HIV cure study?
   3. How do you think other transgender women would feel about stopping HIV treatment for some period of time in order to participate in an HIV cure study?
   4. What are some of the protections researchers should put in place if HIV cure participants are required to temporarily stop their HIV treatment?

**Community Priorities**

1. What are the top priorities for transgender women that you would identify?
2. Considering this list of priorities, how important do you feel it is to find a cure for HIV?

**INTERVIEW TWO**

**Let’s start with talking about the risks and benefits of HIV Cure Research**

1. What benefits do you think there are to participating in HIV cure studies?
2. Do you have any concerns about HIV cure research? If so, what are they?
3. What risks do you think are there to participating in HIV cure studies?
   1. What would be “too much risk” for participants in HIV cure studies?
4. What do you think some of the burdens of participating in HIV cure research are?

**Now, I would like to review the following handout [give participant handout] about HIV cure strategies**

1. How do you feel about what we just discussed?
2. How do you feel about the cure research strategies you heard about?
   1. Ask about strategy A:
   2. Ask about strategy B:
   3. Ask about strategy C:
3. Reflecting on the cure research strategies we discussed, are there any types of HIV cure studies that you would NOT participate in?
   1. What kinds of studies are those?
   2. Why would you NOT participate in these studies?
   3. What would make you more likely to want to participate?
4. Reflecting on the cure research strategies we discussed, are there any types of HIV cure studies that you would WANT to participate in?
   1. What kinds of studies are those?
   2. What appealed to you about those studies?
5. Of the three cure research strategies we talked about, can you rank them from safest to riskiest?
   1. Why do you consider [insert cure research strategy] the safest of the three?
   2. Why do you consider [insert cure research strategy] the riskiest of the three?
6. Tell me about your experience with HIV medications, sometimes called antivirals.
   1. Have you ever taken medicine to treat HIV (providers sometimes call these antivirals)?
      1. If so, what was that like? How do you feel about HIV medicine? What are the good things and bad things about taking the medications? Are you still taking medicine to treat HIV?
      2. If not, tell me about the reasons you have never taken these medicines. What would be required for you to consider taking them?

**Programmatic Considerations**

1. It’s important that all studies are ethical, which means that researchers should do everything they can to protect the people who participate from feeling forced to join a study, from being given wrong information that would cause them to make choices against their best interests, from being exposed to serious harm, and from being mistreated in other ways. Thinking about that, what would an ethical HIV cure study look like or include?
2. Do you think information about HIV cure studies should be promoted to transgender women?
3. What can be done to encourage transgender women to take part in HIV cure studies?
4. What kind of information do you think transgender women who want to participant in HIV cure studies need?
5. What should researchers running HIV cure studies know about working with transgender women?
6. What kind of information do health care providers need about participation of transgender women in HIV cure research?

**Wrap Up and Closing**

Would you like to add anything or make additional comments?
