# Supplementary material for: “I would really want to know that they had my back”: Transgender women’s perceptions of HIV cure-related research in the United States
Source: PLoS One. 2020 Dec 31;15(12):e0244490. doi: 10.1371/journal.pone.0244490 (PMC7774946; doi:10.1371/journal.pone.0244490)
Supplement: S1 Table. Supplemental quotes — (DOCX) [file pone.0244490.s002.docx]

**S1 Table. Supplemental Quotes**

**Transgender Women’s Perceptions of HIV Cure-Related Research: Qualitative Interviews with Transgender Women Living with HIV in Baltimore, MD, United States, January 2020**

| **Themes** | **Quotes** | **Interview** |
| --- | --- | --- |
| **Part 1: Perceptions of HIV Cure-Related Research** | | |
| - 1. **HIV Cure Optimism and Skepticism** | | |
| **Meanings of HIV cure** | | |
|  | *“I mean you will still have to use protection, that's mandatory because it ain't just HIV that's out there. I think that you will have to continue on medications, whatever the medication is that they give you to cure it.”* | PPT 003, PT 1 |
|  | *“I really don’t think about them types of things.”* | PPT 002, PT 1 |
|  | *“What would a cure mean? Extended life for people.”* | PPT 008, PT 2 |
|  | *“Happiness for a lot of human beings that have it.”* | PPT 004, PT 1 |
|  | *“To not have it anymore. Taking some kind of medicine and get rid of the virus forever.”* | PPT 009, PT 1 |
|  | *“It means it's rid of. You're rid of the virus.”* | PPT 003, PT 1 |
|  | *“You'd be able to wipe it clean like it was never here.”* | PPT 008, PT 1 |
|  | *“Cure should be cure, not do this regimen for 5 years or 10 years and hope you survive.”* | PPT 008, PT 1 |
| **Undetectable status vs. HIV cure** | | |
|  | *“Undetectable. Undetectable. I can have unprotected sex and I don't have to worry about passing this disease on to my companion. That's a very good thing.”* | PPT 001, PT 2 |
|  | *“I’m undetectable, but just being undetectable doesn’t mean that you’re cured.”* | PPT 001, PT 1 |
| **Cure optimism** | | |
|  | *“The scientists are getting real, to me, very, very, very close to finding a cure for HIV.”* | PPT 001, PT 1 |
|  | *“Excitement. Anxious. Nerves rattled like, ‘Is it soon? Is it soon? Is it soon?’ It's like that.”* | PPT 006, PT 1 |
|  | *“I mean I guess it just takes time. Hopefully they'll nail it eventually.”* | PPT 003, PT 1 |
|  | *“It could've been fake news or whatever-- it was the UK found a cure, a university over there found a cure for HIV. (…) That was a so excited moment.”* | PPT 006, PT 1 |
|  | *“There is a cure out there. You get enough people together, hopefully, that'll participate in the studies, and eventually there will be a cure for it.”* | PPT 004, PT 2 |
|  | *“I hope and pray that they find a cure. I hope that one day there will be a cure.”* | PPT 005, PT 1 |
|  | *“They're trying to find a cure. They trying to make people lives better if they do come up with a cure. You just have hope and hope and dreams that it is a cure one day.”* | PPT 007, PT 2 |
| **Cure skepticism** | | |
|  | *“I say it’s going to be difficult to find because different people’s bodies are different and levels are different (…) What works for me may not work for another person.”* | PPT 009, PT 1 |
|  | *“I don't think there's a cure. I don't think there'll be a cure in my lifetime.”* | PPT002, PT 1 |
|  | *“I don't think that most of the girls think about it as a possibility in the future.”* | PPT 002, PT 1 |
|  | *“Bull. It's not going to happen.”* | PPT 006, PT 1 |
|  | *“I don't think so. I don't know why that's so hard to-- I mean why is it so hard to figure this out? But I mean I guess it is what it is.”* | PPT 003, PT 1 |
|  | *“I believe that there is a cure, and they know what it is, but they're trying to find a way to make everybody pay for it.”* | PPT 006, PT 2 |
|  | *“I mean I think they're getting close and they're trying real hard, but as of having a complete cure yet? No.”* | PPT 003, PT 1 |
|  | *“It's just sitting there. It's just in there, but it's not affecting you any kind of way (…) You can't pass it through semen.”* | PPT 003, PT2 |
|  | *“And hopefully they come up with the cure, but they probably already have it and they're only giving it out to the wealthy.”* | PPT 009, PT 1 |
| **Perceptions of research** | | |
|  | *“I can't say that I've ever had a negative opinion about it.”* | PPT 008, PT 1 |
|  | *“I think that the initial thing is fear. People fear things that they don't understand. (…) But you have to be willing to learn and open your mind up to different things, and that's what research does.”* | PPT 002, PT 1 |
|  | *“I think it's really important that we constantly learn. We have to know because we're always evolving and, with that, our medicine has to evolve because this disease it changes.”* | PPT 002, PT 1 |
|  | *“Like finding out new ways and new things to improve a situation or to better a situation.”* | PPT 009, PT 1 |
|  | *“A blood draw or a mouth swab or a questionnaire.”* | PPT 008, PT 1 |
|  | *“Just doctors and nurses experimenting to see if they can find a cure, what works better for this person, that person. That's all. That's what comes to mind when you say research.”* | PPT 005, PT 1 |
|  | *“Needles. Probing. Quarantine.”* | PPT 006, PT 1 |
|  | *“A big medical facility with rooms and a whole bunch of IVs and blood and doctors everywhere drawing different people bloods on different levels and testing it.”* | PPT 009, PT 1 |
|  | *“When I thought of research I think of long hallways, white coats, goggles, gloves, needles, drugs and testing and cold shells.”* | PPT 006, PT 1 |
|  | *“We're like guinea pigs.”* | PPT 004, PT 2 |
| **Reasons not to participate** | | |
|  | *“Sometimes just the fear of not knowing, or maybe the fear of knowing.”* | PPT 002, PT 1 |
|  | *“The traveling. The traveling is really bad. Well, for certain people that's outside the area. I'm really not that far (…) Coming doesn't bother me. I actually be excited to come but it's usually just the weather.”* | PPT 002, PT 2 |
|  | *“If it's too far for me and if it's asking for too personal information (…) like, ‘We want to put your name here and put your blood type here, put your [inaudible] number here, your social here, your birthday here, your mom's name here—' that's too personal.”* | PPT 006, PT 1 |
|  | *“A lot of my friends think that their personal information will be shared.”* | PPT 006, PT 1 |
|  | *“Ashamed of what people would think.”* | PPT 008, PT 1 |
|  | *“I would be too scared to try anything new because I'm so hooked on this, the way this treats me.”* | PPT 002, PT 2 |
|  | *“I'm a little set in my ways now when it comes to taking medicines. Actually, my doctor was going to introduce something new to me tomorrow. And I don't know. I'm even on the wall of thinking about that.”* | PPT 002, PT 2 |
|  | *“But if I get a feeling that it's, ‘This is going to affect you,’ I wouldn't do it. And it would be one of those things that I would get all the way to the situation and I would just bail, because I would want to participate, but the thought of what if would be so heavy, I wouldn't be able to do it.”* | PPT 006, PT 2 |
|  | *“We're used to the same routine, but if there's something thrown in there different, a lot of us kind of think should I, or should I not?”* | PPT 004, PT 1 |
|  | *“I want to live with my life just like I'm living it now. I want to keep my ass home, cook dinner and do whatever. But they might have to come and medicate me every day because sometimes I have a problem-- I forget to take my medication sometimes.”* | PPT 001, PT 1 |
| **Reasons to participate** | | |
|  | *“Fine with me. I have no secrets or skeletons or whatever, so I just be fine with whatever.”* | PPT 009, PT 1 |
| **Altruism/service** | | |
|  | *“I wouldn't have no problem in doing a research study because I'm a optimist and I'm open-minded. And especially if it's to a cause to help other people.”* | PPT 009, PT 1 |
|  | *“I'm going here to do this service, whatever, to help. And this new antiviral medicine, this could be better than what I'm taking.”* | PPT 004, PT 1 |
|  | *“I can't have a child, but I think that women who have kids, they will be willing to go and do that research. ‘Do research on me if you can cure this,’ because they want to be there for their kids.”* | PPT 001, PT 1 |
|  | *“Because I try to be careful about the things that people do with my body (…) But I think research is very, very important to help people. I wish-- I mean, I would like to help to research cancer and Alzheimer's because those are things that people I know have been through, (…) It's very sincere in my heart, but I have no problem with research. And I could be a guinea pig with some things, but not for all things.”* | PPT 001, PT 1 |
| **Compensation** | | |
|  | *“For compensation and be cured from the disease, and some to help other people.”* | PPT 009, PT 1 |
|  | *“What’s appealing to me, to be honest, is the gift cards or the cash.”* | PPT 005, PT 1 |
| **Helping advance or enjoying science** | | |
|  | *“I know the reason that I do it is because I get information. I can share information. Like when my girlfriend and I leave, we discuss things.”* | PPT 002, PT 1 |
|  | *“And you learn a lot of things. Like you learn a lot of stuff that you wouldn't know if you wouldn't of went to the study.”* | PPT 006, PT 1 |
|  | *“I just want to learn more and stuff because since I'm dealing with it, I just want to learn more about it.”* | PPT 007, PT 1 |
| **Desire for a cure** | | |
|  | *“I would love to be the one who did the study and they find a cure with me on my study.”* | PPT 003, PT 1 |
|  | *“Well I always like science. And then also, I mean, it is a possibility that somebody is capable of making a cure or something. I would like to be a part of it with them. Help them reach that.”* | PPT 006, PT 1 |
|  | *“That there's a possibility that one of them could work. That they could work. Given the information of how they're looking at it.”* | PPT 009, PT 2 |
| **Care about one’s own life** | | |
|  | *“Because I care about people. I care about my life.”* | PPT 001, PT 1 |
|  | *“But I'm going to go with that new stuff because even with the stuff I'm taking right now, I'm undetectable. But it has not taken the virus out of my system. This new little pill or whatever they've been experimenting and whatever, and it's supposed to take that away.”* | PPT 004, PT 1 |
| **Perceived benefits of HIV cure-related studies** | | |
|  | *“I don't know what the ratio is about how many people is positive or not. But it'd make a big difference to have a cure.”* | PPT 006, PT 2 |
|  | *“I don't know just learning things about it because when you first find out about it you don't know about a lot of things about it and then once you learn things about it, it kind of calms you down some. Kind of calms you down. And you learn how to deal with it and do what you supposed to do.”* | PPT 007, PT 2 |
| **Perceived risks of HIV cure-related studies** | | |
|  | *“You get injected with medicine that make your body have a chemical reaction, I guess. (…) You could be allergic to something and not know it.”* | PPT 008, PT 2 |
|  | *“I have no idea except that it might make some people sick. Well, the after effects. That's it right there. That after effects affect the feelings and if the feelings not right inside, a little monster going to come out. You don't mean for that to come out. You be snapping at people and things.”* | PPT 010, PT 1 & 2 |
|  | *I: People will know that you have HIV.*  *P: Mm-hmm. (…) Everybody. Researchers, participants, whoever the research is on, whoever they link with. Everybody involved, basically.* | PPT 006, PT 2 |
|  | *“The side-effects might be something minor. (…) And after a while, it's gone. You're back to normal. You're taking a med. You're healthy. You're doing the right thing.”* | PPT 004, PT 2 |
|  | *“It'll be minor, compared to what you've got. You may get Eczema or whatever from this medicine. But what's more important: a cure or the Eczema?”* | PPT 004, PT 2 |
|  | *“What's the side-effects? What's the long term that can happen to me because I'm 53 and I'm very cautious on-- I don't need nothing broke [laughter] and I don't need nothing to go array right now.”* | PPT 005, PT 1 |
|  | *“Don't want to get sick. I don't want to go through nothing. I don't want to go through nothing and hurt myself because things can go wrong.”* | PPT 007, PT 2 |
|  | *“You have to worry about my side effects, how my body really works, what my body doesn't like. I guess, a lot of things. (…) they have to know before they start working on somebody because you don't want nothing to happen or something go wrong.”* | PPT 007, PT 2 |
| **Perceptions of unacceptable or ‘too much risk’ in HIV cure-related studies** | | |
|  | *“I would not be okay if it made me bleed, or diarrhea, stuff like that.”* | PPT 008, PT 2 |
| **Perceived concerns about HIV cure-related studies** | | |
|  | *“I really have no concerns. I just wouldn't want it to get any worse [laughter]. That's the only concern that I have.”* | PPT 001, PT 2 |
|  | *“Just concern, hopefully, that they find a cure. So I will do anything to help them get closer and closer.”* | PPT 003, PT 2 |
|  | *“If I had to take medications for it, it would be a concern. (…) Because if it's a study, then it's an experiment. So, I don't want to experiment with my life, because it could be really bad, or it could be really good.”* | PPT 006, PT 2 |
| **Perceived burdens of HIV cure-related studies** | | |
|  | *“No. That wouldn't be a burden for me personally, it wouldn't. No. I would look at it as a little vacation. Like whenever I'm hospitalized with my asthma, I don't be upsetting about it. I take me my little overnight bag, and I gets comfortable in there with the air conditioning and everything. I'll be all right. (…) And there's room service. You can't beat that.”* | PPT 003, PT 2 |
|  | *“One of the burdens is this is experimental medication (…) You don't know the side effects. (…) They could make it worse than what it really is. Because [inaudible] could be like me, HIV positive, undetectable. And once I take that medication, all that goes down the drain.”* | PPT 004, PT 2 |
| **Desirable attributes of HIV cure** | | |
|  | *“I can live with that, if it's something where they say I don't have to take it every day, just take it every 30 days or whatever. That would be perfect.”* | PPT 002, PT 2 |
|  | *“I'm already taking shots in my butt, sugar shots in my hip, sugar shots in finger. No more poking.”* | PPT 002, PT 2 |
|  | *“It all depends. And it all depends on how big them darn pills is. Some of them are just so big they make you gag. And I'm one of those. And if it smell strong with the medicine with it, it makes me sick.”* | PPT 010, PT 1 & 2 |
|  | *“If they had a piece of bubblegum that had the cure for HIV in it, a piece of bubblegum, do you know how many people would be in that line? I could only imagine.”* | PPT 006, PT 1 |
|  | *“Yeah, I don’t like injections. (…) But I’m sure people would take them if it would save their life.”* | PPT, PT 1 |
| **Effects of HIV cure on self** | | |
|  | *“For me at this point, I probably would have a child.”* | PPT 008, PT 1 |
|  | *“My life would still be the same. It just-- I'll just be cured. Nobody don't know nothing.”* | PPT 007, PT 1, SEG 2 |
|  | *“Like if there was a cure it would take months and months for it to get out for everybody, so I don’t think about it. I deal with my reality.”* | PPT 002, PT 1 |
|  | *“If they found a cure for HIV and I was cured and I had my one steady partner, I would never cheat. (…) And then if we broke up-- if our relationship ended, before I had sex with anyone else, I would want both of us-- I would do that; both of us go get tested.”* | PPT 001, PT 1 |
|  | *“And if there's a cure, that great big purple pill, I won't have to ever take it again in my life.”* | PPT 004, PT 1 |
|  | *“I'm still going to be cautious. I'm still going to have safe sex when I do decide to have it. And it's not going to make me run out and say, hey, boys I'm free. No. That's not going to work.”* | PPT 010, PT 1 & 2 |
|  | *“But just knowing that I'm not going to die from that, it would be a good feeling for me personally.”* | PPT 003, PT 1 |
| **Effects of HIV cure on transgender community and others** | | |
|  | *“Well, what could change is people have a longer lasting of life or they'll feel like they have meaning to their life again.”* | PPT 008, PT 1 |
|  | *“They'll be happier. They may not have depression anymore. They may not be so crazy and kill they self or their paths might change. (…) Crazy stuff like doing drugs and degrading they self and selling they self and not educating theyselves.”* | PPT 006, PT 1 |
|  | *“What the cure would mean for me would be so much peace and happiness for so many people.”* | PPT 005, PT 1 |
|  | *“That's a good thing because a lot of people are dying from the disease, so it would be a blessing.”* | PPT 003, PT 1 |
|  | *“Maybe if there's a cure for it, maybe the younger generation will think, "I'm not going to be as promiscuous as they were.”* | PPT 004, PT 2 |
|  | *“We're not going to go into a clinic where it's known that this is where the cure is. No one's going to walk in there. Nobody.”* | PPT 006, PT 2 |
|  | *“It's giving the kids permission to go ahead and have babies again. Oh, my goodness. I never thought of this before, but the population could blow up. It really could. I never thought about that. (…) They could have sex with anybody they want to. They don't have to worry about carrying that particular disease with them.”* | PPT 010, PT 1 & 2 |
|  | *“There'll be a whole lot of whores again over here. I mean, for real. ‘Oh, they got a cure for HIV. You can go fuck whoever you want to [laughter].’”* | PPT 001, PT 1 |
| **Overall impression of HIV cure-related research strategies** | | |
|  | *“Fascinating, fascinating. Oh, my God, it's so fascinating. And everything you're saying is something positive. You did mention about the negative side of it, but most of it's positive.”* | PPT 004, PT 2 |
|  | *“I'm kind of, ‘Yeah, sounds good, but I'm not 21 years old anymore or whatever. Can I deal with this, or whatever, at my age?’ That's a lot to really consider.”* | PPT 004, PT 2 |
|  | *“That's very, very interesting. Very interesting. (…) How I would go about which one I would pick for me.”* | PPT 001, PT 2 |
|  | *“How do they continue to do what they think they should do and never know it might work? Because all of it sounds like it could be possible, like it can work. You know?”* | PPT 009, PT 2 |
| **Perceptions of latency reversing agents** | | |
|  | *“It would be a lot faster, I would think. And I don't think it would be painful. I think that would be a good one. I think that's the one I would pick.”* | PPT 001, PT 2 |
|  | *“I think of it as no survivors. We don't want them hiding [in HIV reservoirs].”* | PPT 002, PT 2 |
|  | *“I'm not a pill popper. It's enough that I've got to deal with the one that I take. I can't. It's just not-- I can't having all those pills.”* | PPT 007, PT 1, SEG 2 |
|  | *“I want to wake up that thing up. The idea is good, really.”* | PPT 001, PT 2 |
|  | *“You might get that big bad one, ‘I ain't going nowhere.’”* | PPT 001, PT 2 |
|  | *“Sounds interesting. And disgusting. These viruses is hiding in your body like that.”* | PPT 009, PT 2 |
|  | *“The kick and kill is basically like I'm going to fight with it inside your body.”* | PPT 008, PT 2 |
|  | *“I mean I wouldn't have an issue with that because it's in there. It's already in there. You know what I'm saying?”* | PPT 003, PT 2 |
| **Perceptions of gene modification and stem cell transplants** | | |
|  | *“That's crazy. Wow.”* | PPT 001, PT 2 |
|  | *“When it's constantly changing, I guess the main thing is to find that one part of it that can be attacked.”* | PPT 002, PT 2 |
|  | *“Because you can learn about the genes, you can know more about the people's body or whoever the patient is. That way you can work your way into knowing how to treat things. That seems to be easier and less complicated.”* | PPT 010, PT 1 & 2 |
|  | *“That sounds more logical to me or whatever, because we all have DNA's or whatever. And like you said, they can't get through here or whatever.”* | PPT 004, PT 2 |
|  | *“Have to rebuild your immune system.”* | PPT 007, PT 2 |
|  | *“It needs something to feed on. So if you get rid of this thing that it needs to feed on, then that's a step closer.”* | PPT 002, PT 2 |
|  | *“That's a good idea. But it sounds like it's so torturous and painful.”* | PPT 001, PT 2 |
| **Perceptions of therapeutic vaccines** | | |
|  | *“And they shoot it into you and they see if your body is strong enough to fight it off.”* | PPT 008, PT 2 |
|  | *“I don't get it-- some woman had got it and was on this and she's healthy and an athlete and it crippled her. (…) It crippled her and paralyzed her or something. (…) With the flu shot.”* | PPT 009, PT 2 |
|  | *“Because with most vaccines, they usually have a counter vaccine. (…) So if something goes wrong, they are able to at least put you back to where you were before they started doing what they were doing.”* | PPT 002, PT 2 |
|  | *“It could be just the word, that I'm comfortable with the word vaccine because when I think of vaccine, I think of cure. I think of health. So vaccine doesn't sound scary to me.”* | PPT 002, PT 2 |
|  | *“With the virus, you're infecting me and possibly giving me something worse than I might already have.”* | PPT 008, PT 2 |
|  | *“So vaccine doesn’t sound scary to me. So it would definitely be—that would be why I would probably choose—I’m able to choose vaccine.”* | PPT002, PT 2 |
| **HIV cure-related strategies respondents would not participate in [topic not in manuscript]** | | |
|  | *“The one I would not chance is probably the one where the HIV is somewhere in my body, hidden. (…) Yeah, Kick-and-Kill.”* | PPT 004, PT 2 |
|  | *“I'm not a pill popper. It's enough that I've got to deal with the one that I take. I can't. It's just not-- I can't having all those pills.”* | PPT 007, PT 1, SEG 2 |
|  | *“But if it's like surgery type stuff or like foreign substance injections and all that, no.”* | PPT 009, PT 1 |
|  | *“Because it's something I don't think they're really sure of. They're not really sure and it's like-- they still have work to do on all of them. But on that one, I think they have more work to do.”* | PPT 010, PT 1 & 2 |
|  | *I: So, potentially, they could all involve needles of injecting something into your system.*  *P: I can't do it.* | PPT 002, PT 2 |
|  | *“Now getting cut open, not doing it. (…) Getting long needles stuck in my back. Not doing it. (...) And radiology, going under the radiation, I'm not doing.”* | PPT 009, PT 2 |
|  | *“If not a medical reason, ain't nobody putting a needle in my arm, nowhere. No. I don't like needles. I definitely don't like needles.”* | PPT 010, PT 1 & 2 |
| **Part 2: Perceptions of HIV Treatment Interruptions** | | |
| - 1. **Mixed Perceptions of HIV Treatment Interruptions** | | |
| **Perceived advantages of HIV medications** | | |
|  | *“And there are no side-effects that they have, as I was saying. And then after a while I'm-- I do notice this. Maybe it's just me. It gives me a little bit of pep.”* | PPT 004, PT 2 |
|  | *“This pill is helping you, too. That pill is your lifeline, and you should be happy to take it.”* | PPT 004, PT 2 |
|  | *“I can't say, ‘Oh, it'll make you do this. You can see this. You can feel that.’ No. All I can say is just, it gave hope. It's, ‘Okay. I'm not going to die today. I've got stuff.’”* | PPT 006, PT 2 |
|  | *“And I've been undetected twice, but this has been the longest since I've been non-detected, but it's been off of the medication. Off the Genvoya. And it doesn't make me sick. It doesn't cramp my stomach.”* | PPT 006, PT 2 |
|  | *“It's fine. I just take one little pill. One little pill. I'm fine. Everything is good when I go to (…) the doctor, sorry. Everything is good. I take care of myself very well. I don't do that unprotected-- I just don't. I'm just fine. It's just take your medicine and you'll be fine. Just do what you have to do.”* | PPT 007, PT 2 |
|  | *“Medicine got better because back in the '80s and the '90s it was more people dying of it. But now the medicine have got better, people living longer.”* | PPT 007, PT 2 |
| **Perceived drawbacks of HIV medications** | | |
|  | *“They make you drowsy. They make your stomach hurt. Oy, but they make you want to over-concern. (…) Like whatever that you see that you could possibly eat it, that's how you going to eat it whether you're hungry or not.”* | PPT 008, PT 2 |
|  | *“It's like a reality check every time I go to take my medicine. It's a one pill a day, but every time I take it it's like a reality check like, ‘Uh. Here we go again. It's coming back. Remember me?’”* | PPT 006, PT 1 |
|  | *“I remember when this disease first arose. How many people died from the medications that they were taking and-- what was that first one? AZT or something like that? How many people passed away. And how the medications have really advanced.”* | PPT 001, PT 2 |
|  | *“I remember when pills first came out, and they were making people giving you big stomachs and bloating up certain parts of your body. And that was my biggest fear. I was like, I didn't want that big pot belly.”* | PPT 002, PT 1 |
|  | *“Just that I have to take it just because of the situation of me having it.”* | PPT 003, PT 2 |
| **Willingness to interrupt HIV treatment** | | |
|  | *“I would do it. I would do it. I would do it.”* | PPT 001, PT 1 |
|  | *“I mean if it was a paid study, okay. I believe so.”* | PPT 006, PT 1 |
|  | *“Yeah, because you're still being monitored in hospital.”* | PPT 008, PT 1 |
| **Perceived concerns about HIV treatment interruptions** | | |
|  | *“Because if you gave somebody something that you could just take out at home, it takes forever for the ambulance to get there, and you don’t know if the person going to be able to get to the phone. Then they could die on their way to hospital.”* | PPT 009, PT 1 |
|  | *“So the medication I was taking could really get out of my system and I'm putting a new medication into my system because I wouldn't want to have-- I don't know, they might clash. I might end up being fucked up.”* | PPT 001, PT 1 |
|  | *“Is it going to take away from my energy? Is it going to give me energy? I mean, I just have so many variables that I think about.”* | PPT 002, PT 1 |
| **Part 3: Considerations for Transgender Women and HIV Cure-Related Research** | | |
| **Competing Priorities for Transgender Women** | | |
|  | *“I think that a lot of people wouldn't really care if they got [an HIV cure] because they wouldn't be careful. They'd go, ‘I can go to the [inaudible] and take this pill and stuff.’”* | PPT 001, PT 1 |
|  | *“Not on my list, no.”* | PPT 002, PT 1 |
|  | *“Me personally, it's not [a priority] -- Whether they find it or not, I'm still living my life.”* | PPT 008, PT 1 |
|  | *“Yes, it is. Yes, it is. Yes, it is. But I think a lot of people would just think that it was just another venereal disease and that’s it.”* | PPT 001, PT 1 |
| **Necessary protections for HIV cure-related studies** | | |
|  | *“I think you just have to be honest of what could happen and what couldn't happen, and how safe and unsafe this research is, and give a person an open door to-- whether they want to do it or not.”* | PPT 005, PT 1 |
|  | *“They could call me. (…) And also, ‘How do you feel? Do you feel better now that you're taking this new one compared to the old one? What's the difference between the two? ...’”* | PPT 004, PT 1 |
|  | *“I would hope they would explain to me what the side effects are of each medication that I'm taking or whatever.”* | PPT 003, PT 1 |
|  | *“I would want to know what’s going on with the research as far as me, individually--"* | PPT 002, PT 1 |
|  | *“Like if they put me on another medicine, and I'm not satisfied with it. Or it's not given them the results that they were looking for. Would I then be transferred-- be able to go back to my original medicine that was putting me where I needed to be.”* | PPT 002, PT 1 |
|  | *“Yeah, support me, make sure that my blood is clean from the medications I was taking before I got put on a new medication (…) I would be a person that would be willing to try a new medication. Just make sure the old medication is out of my system before you give me a new medication.”* | PPT 001, PT 1 |
|  | *“I would think that you would offer - and you should offer - if you get this side-effect we can take care of you until it gets better. Or if you start having an adverse reaction to any of the medication and you don't-- we'll be there to be there to help you get better. (…) We don't want to leave people left dealing with the after-effects of research that's trying to help people. It's just not the human thing to do.”* | PPT 005, PT 1 |
|  | *“I think as long as they made sure my health was up to par. Then I would have no problem. I would have absolutely no problem. But I just, like I said, I like to be in the loop.”* | PPT 002, PT 1 |
|  | *“But as long as you can show me proof that this is going to work, and I won't grow another eyeball.”* | PPT 002, PT 1 |
|  | *“Have a medical team on standby, like right there, so if I’m going through something or something happens, they can help get me out of it.”* | PPT 009, PT 1 |
|  | *“I would love to hear someone else went through it and they've successfully became HIV negative.”* | PPT 007, PT 2 |
|  | *“And to have ethics, I guess would be to have a certain common decency, or upholding about yourself being. (…) Making a patient, or whoever's doing the study is as comfortable as possible. (…) I would guess showing that you care, or basically asking them do they need something. It's the little things that make a big difference.”* | PPT 008, PT 2 |
|  | *“You're taking a chance by going in the study. (…) So I mean just treating me with respect and just do what you're supposed to do. That's the only thing I can say about it.”* | PPT 003, PT 2 |
|  | *“It's do what they want. What they want to. Make their own decisions. And not feel timid or feel like it's something that they have to do. And let them know they have a option to do it or not to do it.”* | PPT 009, PT 2 |
|  | *“Have evidence that what they're trying to do has a possibility of it working (…) Evidence. Okay? Not, ‘I'll take it and try this one, and then try this one, and try this one.’ Nope. (…) I don't want to go through that.”* | PPT 006, PT 2 |
| **Considerations specific for transgender women** | | |
|  | *I: What do you think other trans women know about HIV cure research?*  *P: Very little because they don't get into it. They don't like exposing themselves. And it's just that whole-- a lot of people don't feel comfortable exposing their status. (…) Because of what others might think.* | PPT 009, PT 1 |
|  | *“It should be promoted to everybody, because we're not only ones that carry it, but we get a bad name. Well, not a name. A bad rap. We're all known as sex workers. ‘Give it to the whores. Give it to the prostitutes.’ I can see a lot of people would say that.”* | PPT 006, PT 2 |
|  | *“Basically that you working with a human, and trans is just a title.”* | PPT 008, PT 2 |
| **Information for transgender women** | | |
|  | *“Oh, send them a newsletter to their house if they want. If they're willing to accept (…) It's something that will keep the public informed. I think that would be a very good idea.”* | PPT 001, PT 2 |
|  | *“All information that they need to make the clients or the patients feel comfortable and know everything there is to know about what they getting theirself into.”* | PPT 009, PT 2 |
|  | *“Oh, gosh. What information would I like? I would like to know all of it.”* | PPT 001, PT 2 |
|  | *“We need all the information we can get. Especially these young girls because they just are, oh. They terrible.”* | PPT 001, PT 2 |
|  | *“Well they would need to know basic research. I mean, basic information about it.”* | PPT 008, PT 2 |
|  | *I: Do you think researchers should be promoting information about HIV cure studies to trans women?*  *P: Yeah. To everybody. (…) So everybody can benefit off it in some type way. Now those that are infected, they benefit out of it more than those that are not. But it's still the information.* | PPT 009, PT 2 |
|  | *“Or my first thought was a YouTube video (…) Write down secures, genotypes, like it's one thing to literally sit here and have a discussion about this is this, this is this. But if you could see firsthand like okay, this is gene therapy, taking this blood, going here, and inject in this. This is this. You only have so much to think about or try to imagine what's actually going on.”* | PPT 008, PT 2 |
| **Strategies to encourage participation of transgender women** | | |
|  | *“Men. For really, for real. Say there's going to be a stripper party or something. And all the girls, they'll show up for that. And it'd just be one big meeting or something. A lot of them might leave. But that's one way to get us out.”* | PPT 001, PT 2 |
|  | *“Talk to them. Because sometimes you go to go to different places or go on the internet and you see a tran, hit them up, send them all things, send thing through emails, go to groups where they're at, go to clubs where they're at.”* | PPT 007, PT 2 |
|  | *“The more the merrier. But the less public it is, the better it is.”* | PPT 006, PT 2 |
|  | *“The focus groups or whatever, they're good. I like them. (…) you get a group of girls in a room, and you start feeling and seeing real emotions and real rawness in people. (…) it's mind-blowing that, as trans women, we have all been down that same road, majority of hurt, pain, disease, whatever it may be.”* | PPT 002, PT 2 |
|  | *“Call them on the phone, and have a conversation with them. Now, hopefully - not in person, over the phone - convince them that this is the new millennium. These are things that are still in research, but these are things to help you. (…) ‘You need to come in one-on-one with me? Don't hide behind the phone. Come up here and talk.’ (…) On the phone, you can hide your emotions from me.”* | PPT 004, PT 2 |
|  | *“I really can't say because some people is so private about their lives and stuff.”* | PPT 007, PT 2 |
|  | *“I really don't know because they done tried with the money, they done tried with the food. [Name], we tried having a Christmas party and bought all these gifts just to give if they come to the party free. The food and everything, everything's free. All you have to do is come to the party. And we just communicate. I think we got five people.”* | PPT 010, PT 1 & 2 |
